# Supplementary material for: Discovery of a non-canonical prototype long-chain monoacylglycerol lipase through a structure-based endogenous reaction intermediate complex
Source: Nat Commun. 2023 Nov 27;14:7649. doi: 10.1038/s41467-023-43354-4 (PMC10682391; doi:10.1038/s41467-023-43354-4)
Supplement: Supplementary file 1 — Supplementary Information [file 41467_2023_43354_MOESM1_ESM.pdf]

Supplementary Figures

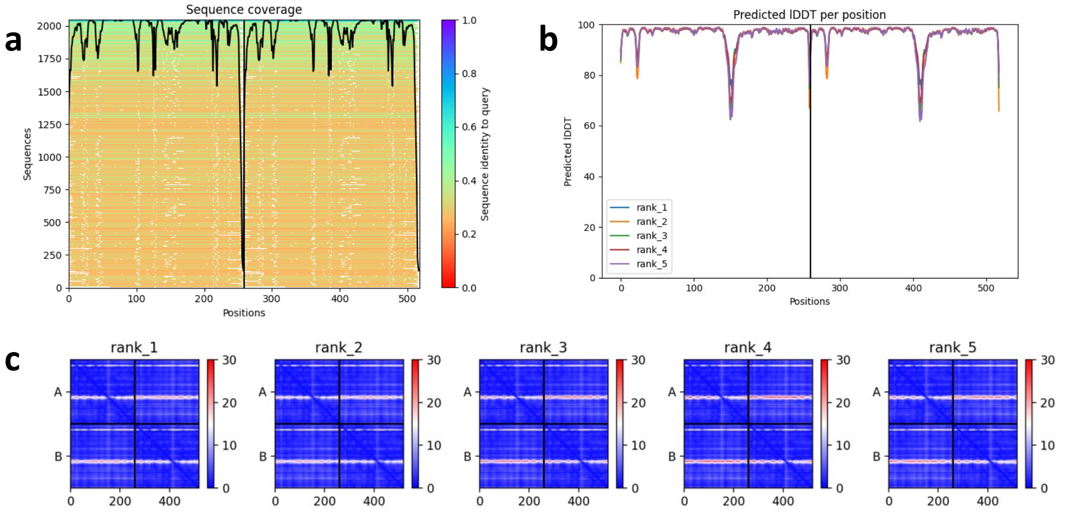

Supplementary Figure 1. Alpha Fold 2 predicted structures of dimeric *Tth* MAG lipase The overall root mean square deviations of the five top predicted structures with the experimental *Tth* MAG lipase structures are in the range of 0.4-0.5 Å. The majority of the *Tth* dimer interface specific interactions **(Fig. 2c)** are not recovered in the predicted structures. In addition, any information on protein-ligand interactions (**Fig. 3**) is missing in the predicted models. The vertical and horizontal lines shown in the plots indicate the border between the first and second sequence chains (A, B) of the predicted models. **a**, Sequence coverage of the predicted models; **b,** Local Distance Difference Test, indicating a major peak within the sequence segment of the *Tth* MAG lipase lid domain; **c,** Distance matrices of the top five ranking models, indicating the highest level of uncertainty at the sequence segment of the *Tth* MAG lipase lid domain.

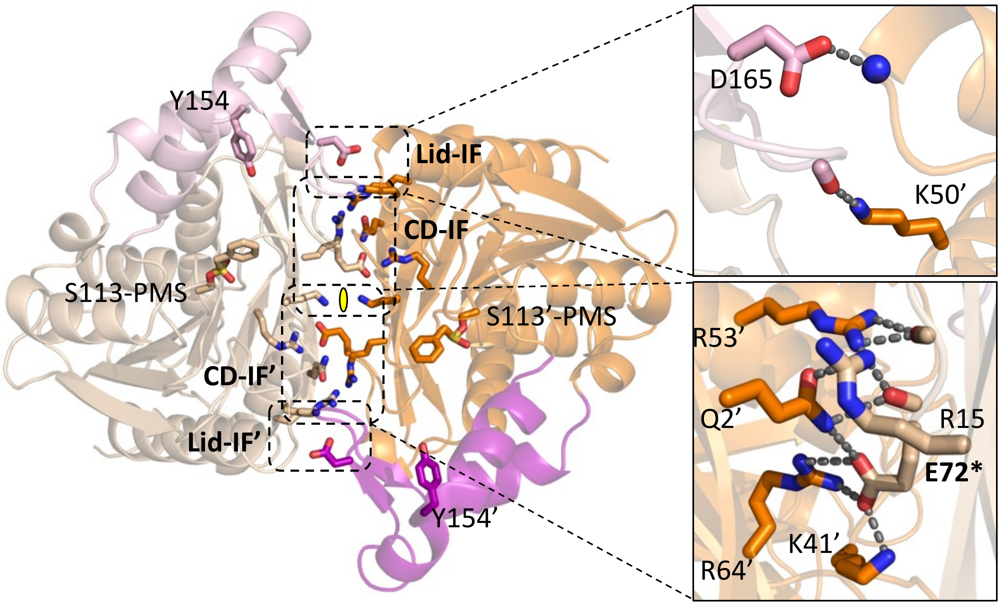

Supplementary Figure 2. Two-fold symmetric dimeric interface of *Tth* MAG lipase. Two-fold symmetry is indicated by a yellow symbol. The majority of specific interactions originate from the two-fold repeated central CD interface labeled CD-IF and CD-IF', and a smaller number of interactions originate from the two-fold repeated distal lid domain interface labeled Lid-IF and Lid-IF'. Zoom-in of the interfaces are shown on the right indicating specific hydrogen bonds (cf. panel D, Supplementary Figure S2). As the PMSF-inhibited *Tth* MAG lipase structure is shown, Ser113-PMS is labeled in both protomers, to approximately locate the two active sites. The position of Tyr154, which was used for mutational analysis, is also labeled. Each *Tth* MAG lipase molecule comprises a two-domain structure. The larger CD is assembled by two sequence segments (residues 1-140 and 184-259), which form a well-established α/β-hydrolase fold comprising a central eight-stranded superhelical twisted β-sheet and six α-helices positioned on both sides of the sheet. In the PMSF-inhibited *Tth* MAG lipase structure, a covalent PMS-adduct with the catalytic Ser113 is formed, thus blocking the active site from further enzymatic reaction ligands (**Fig. 3B**). In agreement with other lipase structures, *Tth* MAG lipase Ser113 is located at the nucleophilic elbow directly after strand β5 to act as a catalytic residue adopting a strained ε-conformation **(Fig. 3a-c, Supplementary Fig. S3**). This position is part of the catalytic triad, which also includes Asp203 and His233 **(Fig. 3a-b, Supplementary Fig. 2).** In both structures an active oxyanion hole is formed by Phe37 and Met114 by orienting their main chain amido groups towards the MAG lipase active site **(Fig. 3b).**

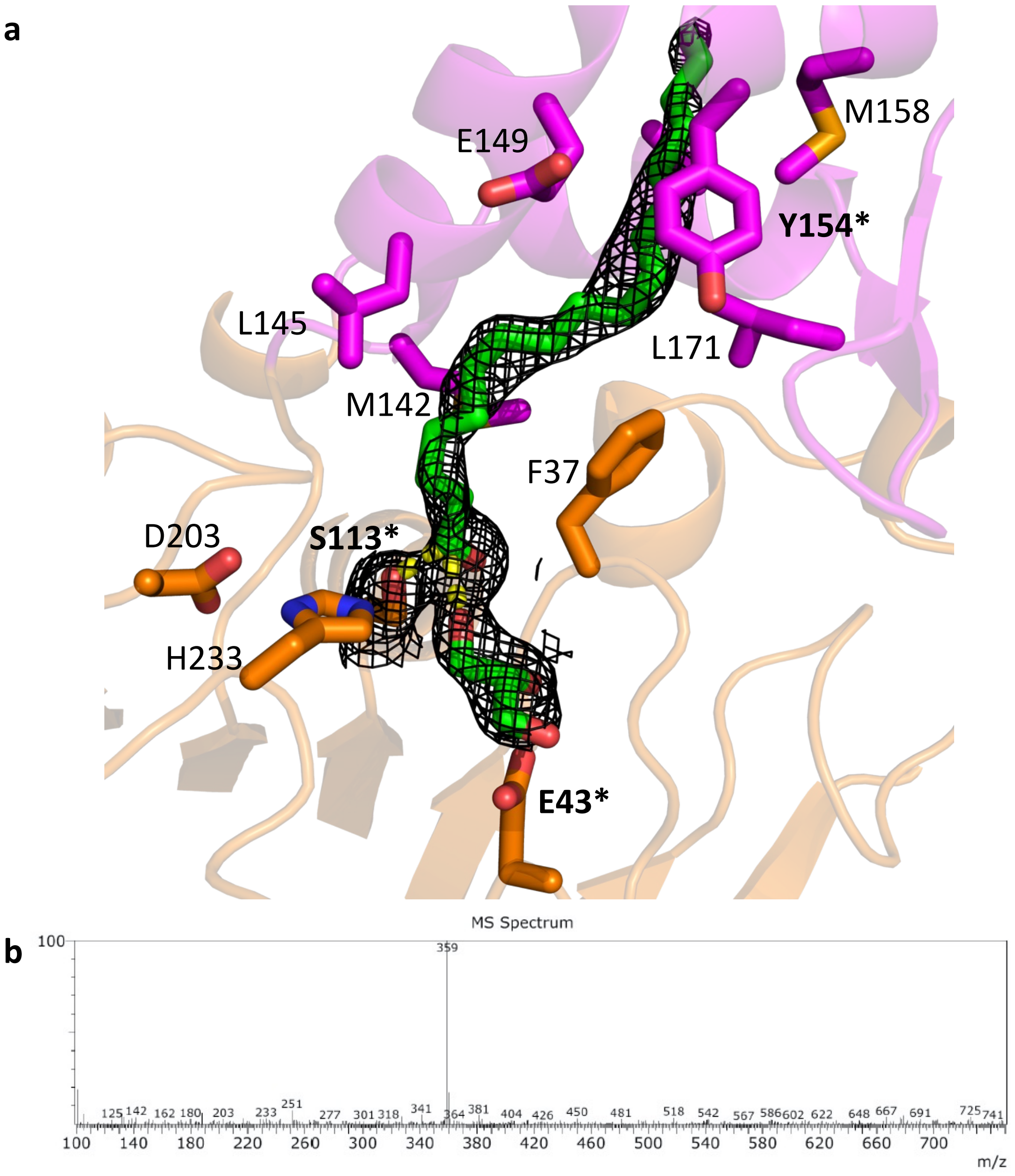

Supplementary Figure 3. a, evidence for the presence of endogenous C18 MAG intermediate in the *Tth* MAG lipase structure without PMSF. *2Fo-Fc* omit electron density map at 1 σ contour level. *Tth* MAG lipase residues that are involved in binding to C18 MAG intermediate are shown and labeled (*cf*. **Fig. 3a**). **b,** reverse phase chromatography analysis of bound ligands in *Tth* MAG lipase crystals in the absence of PMSF**.** Source data are provided within **Supplementary Data 2.**

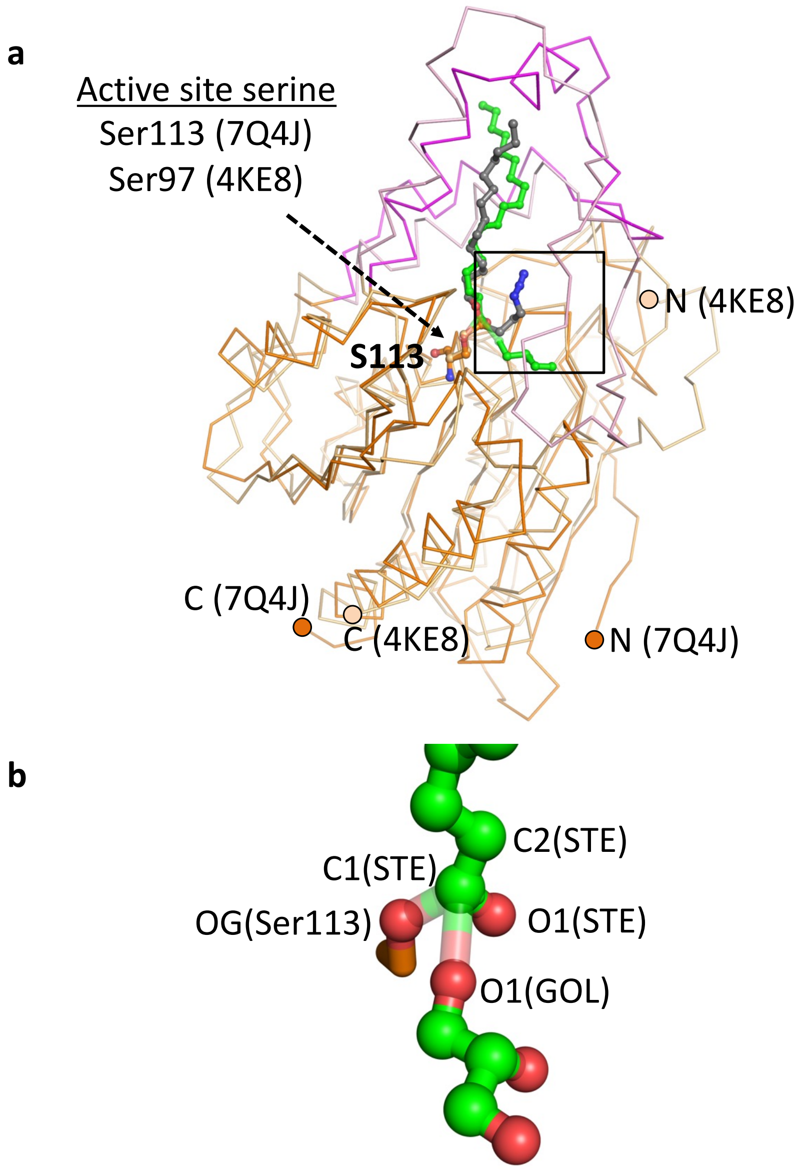

Supplementary Figure 4. Geometry of MAG intermediate in *Tth* MAG lipase. a, superposition of *Tth* MAG lipase in complex with C18 MAG intermediate and *Bacillus sp.* MAG lipase in complex with C14 MAG analogue (PDB entry: 4KE8). Colors for the *Tth* MAG lipase complex are as in **Fig. 1** and **2.** Colors for *Bacillus sp.* MAG lipase in complex with C14 MAG analogue: protein, light orange; MAG analogue, grey. Oxygen and nitrogen atoms are in red and blue, respectively. **b**, zoom into *Tth* MAG lipase MAG tetrahedral intermediate. Transient bonds are in pale colors. Atoms are specified as in **Supplementary Table 11.**

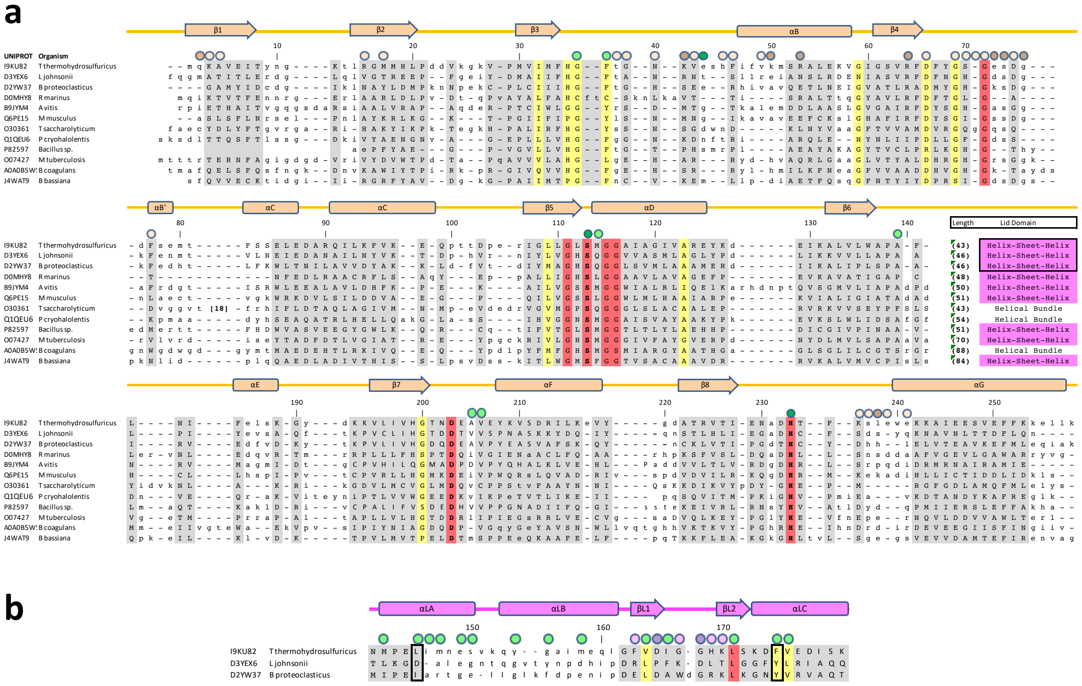

Supplementary Figure 5. a, alignment of 12 CD sequences, for which an overall structure-based alignment was possible. UNIPROT codes and organisms of aligned sequences are indicated to the left. Structurally aligned residues are shown in capital letters and respective aligned positions are shaded in grey; other residues are in small letters. As a structure-based alignment of the lid domain segment, inserted into the N-terminal and C-terminal part of each CD sequence, was not possible due to high level of sequence and structural diversity, instead the length and type of lid domains of all chosen esterase/lipases are indicated. HBH-type lid domains with structural relations to the *Tth* MAG lipase lid domain are colored in magenta. Sequence numbers indicated in 10-residues intervals on top of the alignment refer to the *Tth* MAG lipase sequence. Helices and β-strands, indicated by cylinders and arrows, respectively, are taken from the *Tth* MAG lipase coordinates. The coloring codes of these secondary structural elements are as in **Fig. 2**. Long insertions of single sequences are shown by numbers, indicating the length of the inserted sequence. Conserved residue positions are highlighted in red (invariant) or yellow (highly conserved). Residues involved in ligand interactions (C18 MAG) in the *Tth* MAG lipase structure without PMSF are indicated by green circles on top of the alignment: hydrogen bond interactions, dark green; other interactions, light green. Residues involved in *Tth* MAG lipase dimer interactions are indicated by orange circles: hydrogen bond interactions, dark orange; other interactions, light orange. **b,** alignment of the HBH lid domains of three two sequences closely related to *Tth* MAG lipase, for which a structure-based alignment was possible. The color codes are as for panel a, except residues involved into lid domain dimer interface interactions are highlighted with magenta circles according to the color codes from **Fig. 2** on top of the alignment. Residues with changed properties for active site ligand binding are boxed.

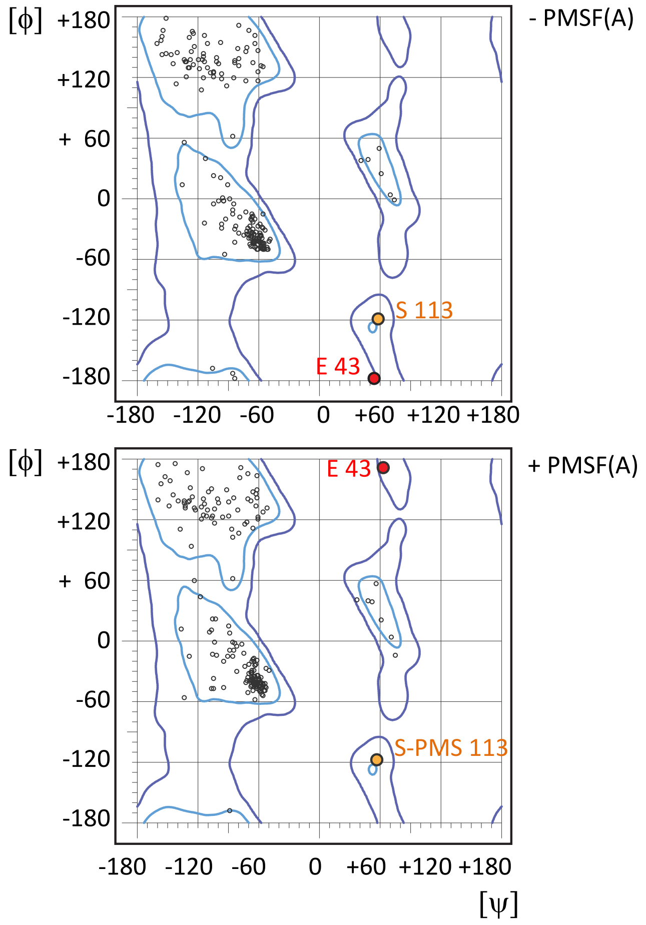

Supplementary Figure 6. Ramachandran plot of *Tth* MAG lipase structures, in the absence and presence of PMSF. In both structures, Glu43 and Ser113 have unusual dihedral angles, due to functional reasons (for details, see text).

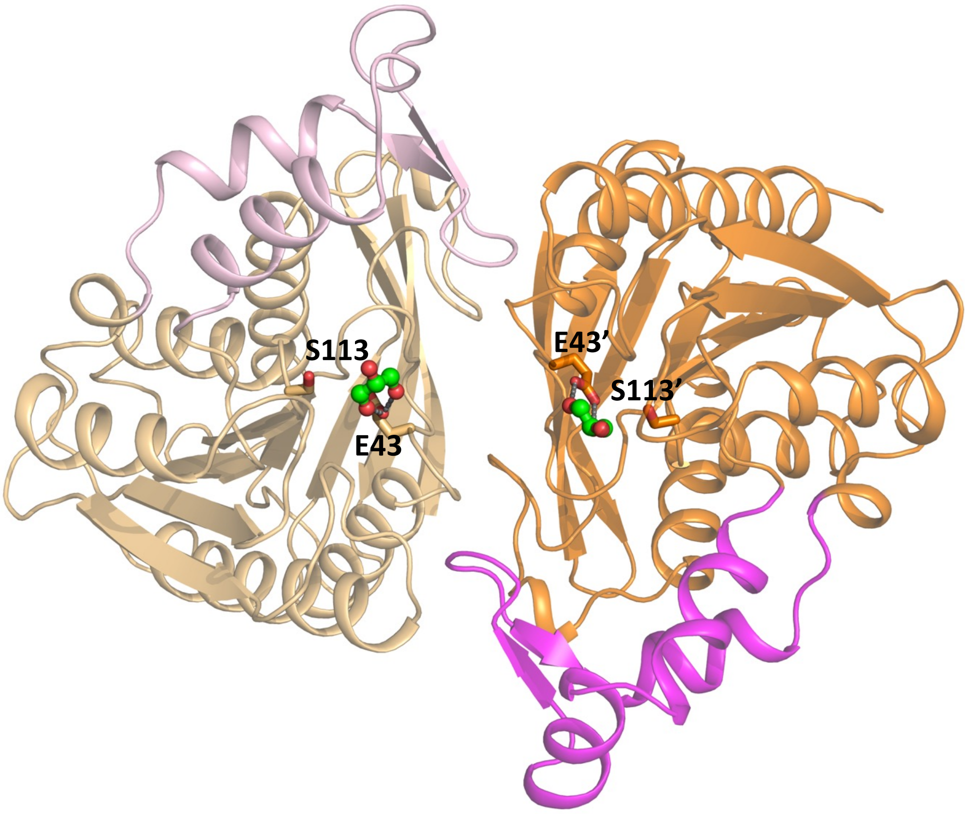

Supplementary Figure 7. Structure of native, non-methylated *Tth* MAG lipase (PDB code: 8B9S), demonstrating the presence of glycerol in the *Tth* MAG lipase active site. Color codes are as in **Fig. 1** and **2.** In contrast to the structure of methylated *Tth* MAG lipase (PDB entry: 7Q4J) there is no evidence for an acylglyeride intermediate.

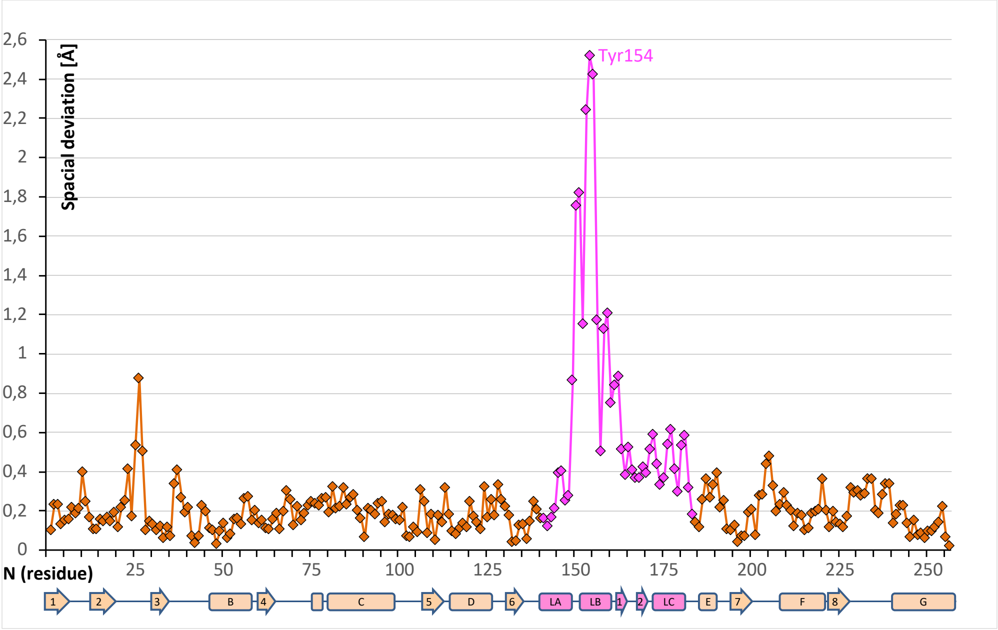

Supplementary Figure 8. Dynamic substrate tunnel opening in the *Tth* MAG lipase lid domain (*cf*. **Fig. 3D-E**). Spatial differences of the Cα positions of the *Tth* MAG lipase structures, with and without PMSF. Color codes are as in **Fig. 2.** The approximate positions of secondary structural elements are indicated **(Fig. 2a, Supplementary Fig. 2)**. The comparison indicates peak movements of lid domain residues next to the tunnel opener Tyr154.

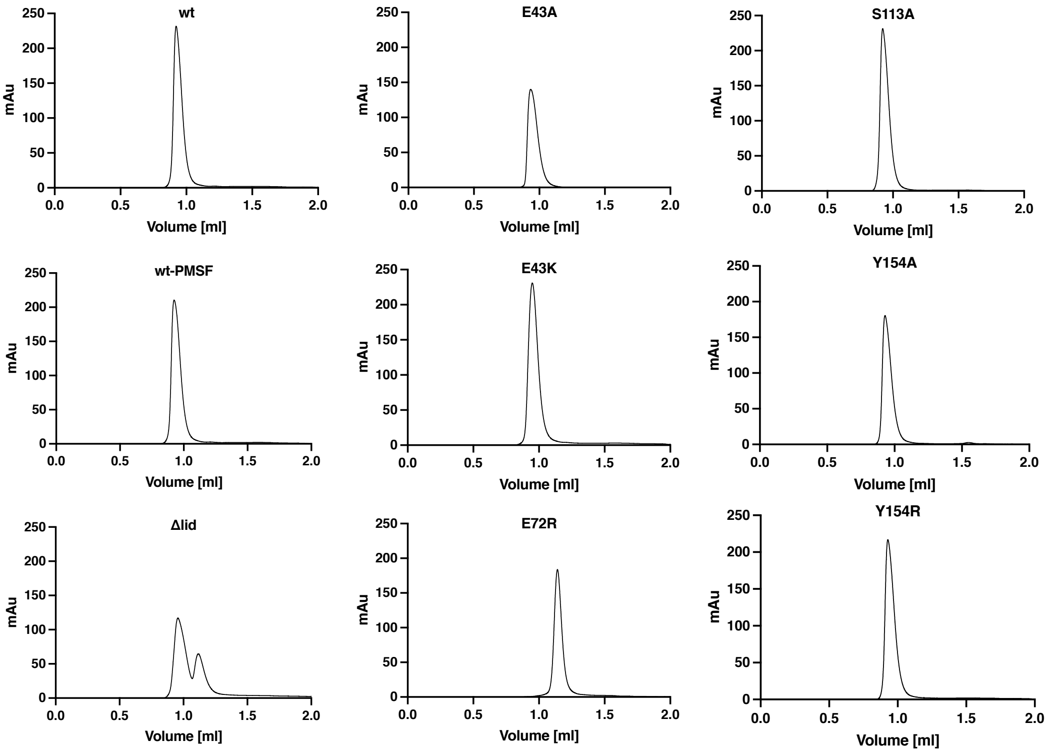

Supplementary Figure 9. Analytic SEC profiles of all *Tth* MAG lipase variants investigated. The respective elution profiles – except the ΔLid and E72R mutants – were interpreted as showing a dimeric state of the enzyme. Detection has been in milli Absorbance Units [mAU]. Source data are provided as a Source Data file.

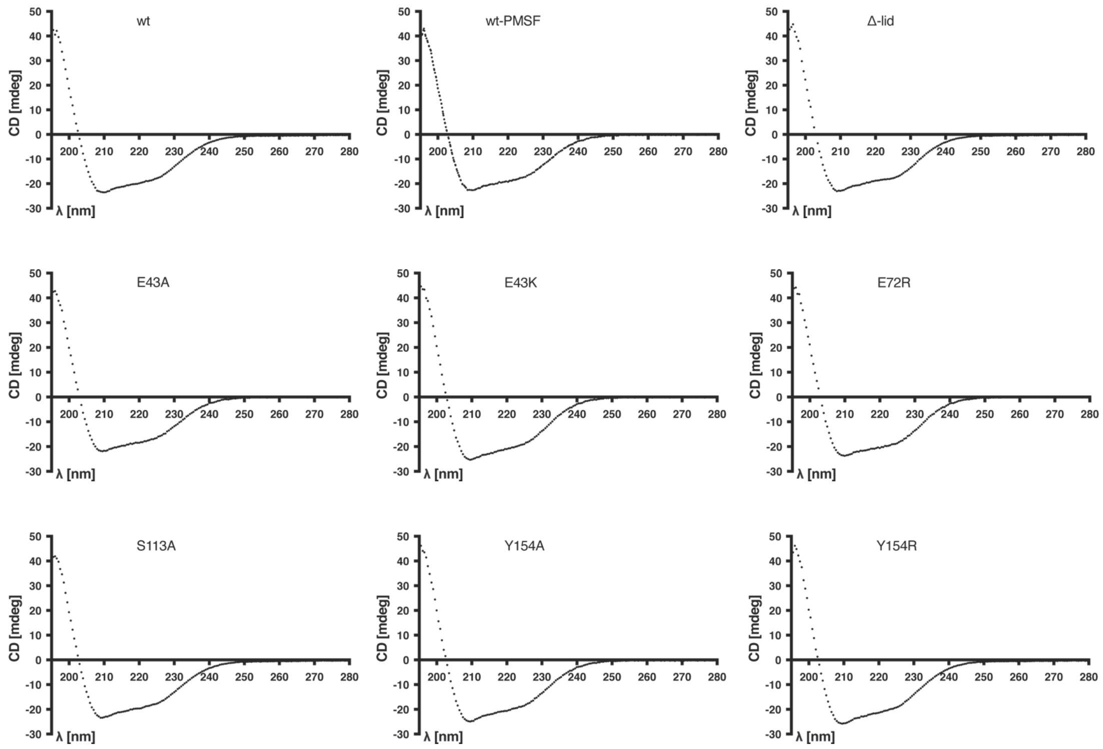

Supplementary Figure 10. CD spectra for all MAG lipase *Tth* variants investigated, showing no significant deviations from wt *Tth* MAG lipase. Samples were measured in triplicates. For quantification of secondary structure content, see **Supplementary Table 4.** Source data are provided as a Source Data file.

a

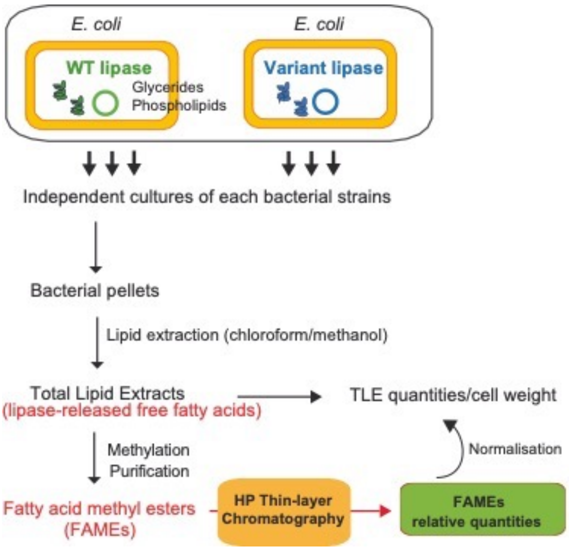

b

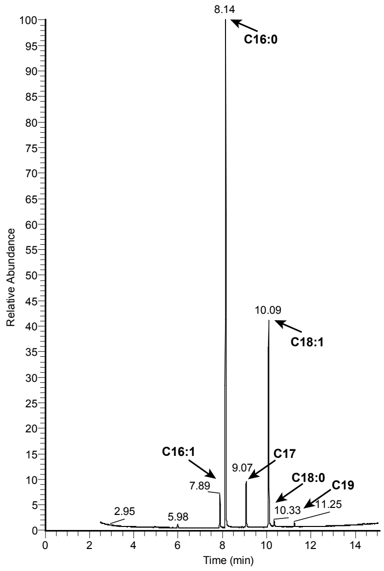

Supplementary Figure 11. a, Schematic workflow of sample preparation and lipid cell-based data analysis. *E. coli* cultures were transformed with *Tth* MAG lipase variants, induced with IPTG for 60 min. Bacterial pellets were used to prepare total lipid extracts (TLE) following chloroform/methanol extraction. Lipid extracts were methylated to yield Fatty Acid Methyl Esters (FAMEs), further purified though florisil columns and the resulting enriched FAMEs were analyzed by high-pressure thin-layer chromatography (HPTLC) and quantified. Plate visualization was realized using primuline dye and scanning. Fluorescence associated with FAME bands was quantified, and normalization to TLE and bacterial mass was performed to correct for variations across samples. **b,** representative gas chromatogram used for FAME analysis.

a

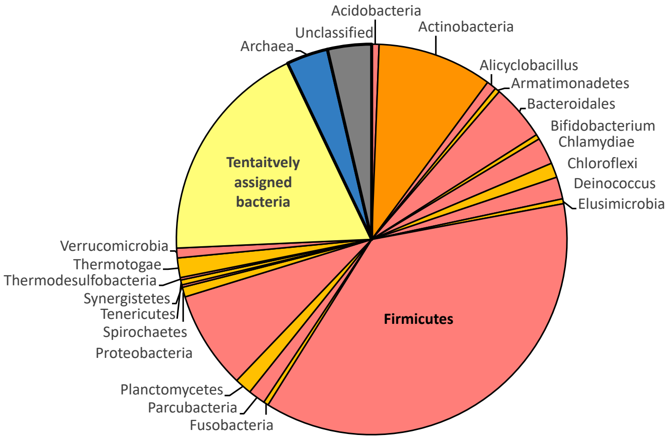

.

b

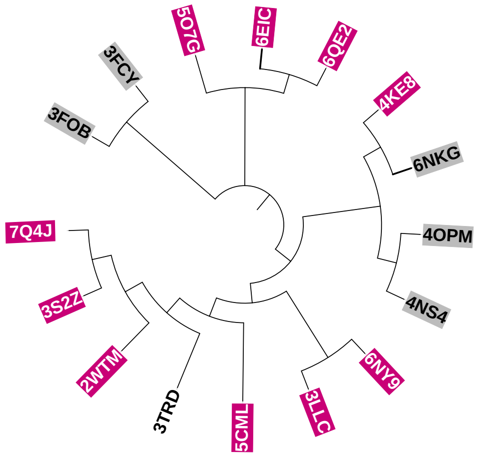

Supplementary Figure 12. a, taxonomic analysis of most closely related 496 sequence groups as defined in UniprotKB50, indicating that all related sequences (to the extent unambiguously assigned) are either of bacterial or archaeal origin. The statistics for different bacterial phyla are shown in alphabetic order and in alternating colors (red, orange). Statistics of unclassified or tentatively assigned bacterial sequences are shown in yellow, archaeal sequences in blue, and unclassified sequences are in grey. The complete list of sequence groups is in **Supplementary Data 1**. **b,** phylogenetic tree presentation of sequences with known 3D structures and distant relations to *Tth* MAG lipase, indicating the respective PDB ID codes (*cf***. Supplementary Table 8**). Sequences containing a HBH lid domain are highlighted in pink **(*cf*. Fig. 7),** other sequences with helical lid domains are in grey. The precise phylogenetic distances are not reliable because of only distant relations and hence have no meaning in this presentation. Conditions for target selection: a) presence of a complete α/β hydrolase catalytic triad (Ser113, Asp203, His233 in the *Tth* MAG lipase sequence) to discard any false-positive hits with unrelated function, b) coverage of both the N-terminal and C-terminal segments of the *Tth* MAG lipase domain with the lid domain inserted in the resulting pairwise sequence alignments.

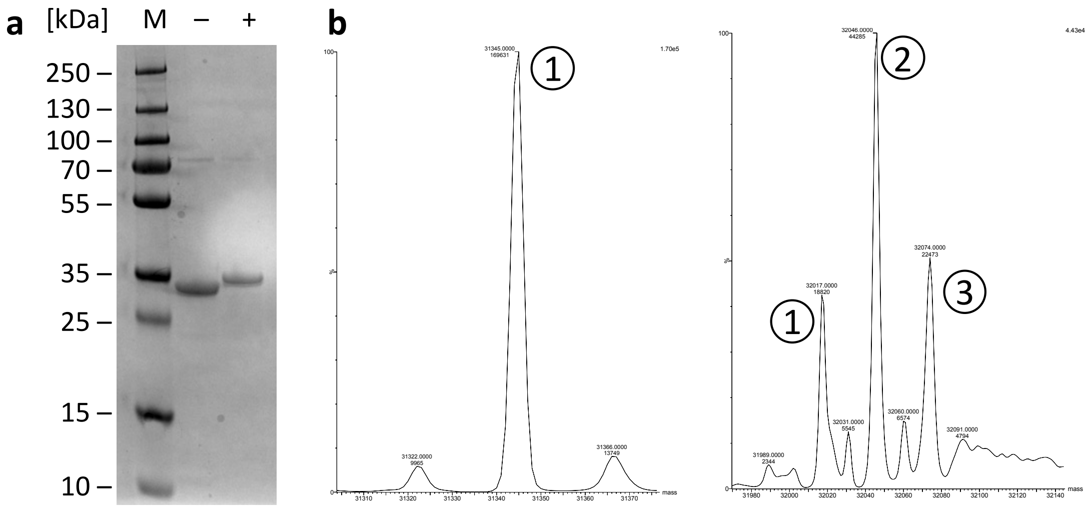

Peak	Native *Tth* MGL [Da]	Methylated *Tth* MGL [Da]	Difference [Da]	# Methylation Sites
1	31345	32017	672	24
2		32046	701	25
3		32074	729	26

Supplementary Figure 13. Evidence for *Tth* MAG lipase methylation. **a**, SDS-PAGE. M, marker; –, native *Tth* MAG lipase; +, methylated *Tth* MAG lipase. **b,** intact mass spectrometry: left, native *Tth* MAG lipase; right, methylated *Tth* MAG lipase. Measured molecular masses are listed below. The measured molecular mass of the native *Tth* MAG lipase includes the sequence of the N-terminal poly-histidine tag (MKHHHHHHSAGLEVLFQGP). Lysine di-methylation adds 28 Da per site, or when deprotonated 29 Da per site. The experiment was carried out once. Uncropped image of panel a) is provided in Source Data file. Source data from panel b) are provided within SourceDataPDF.zip file.

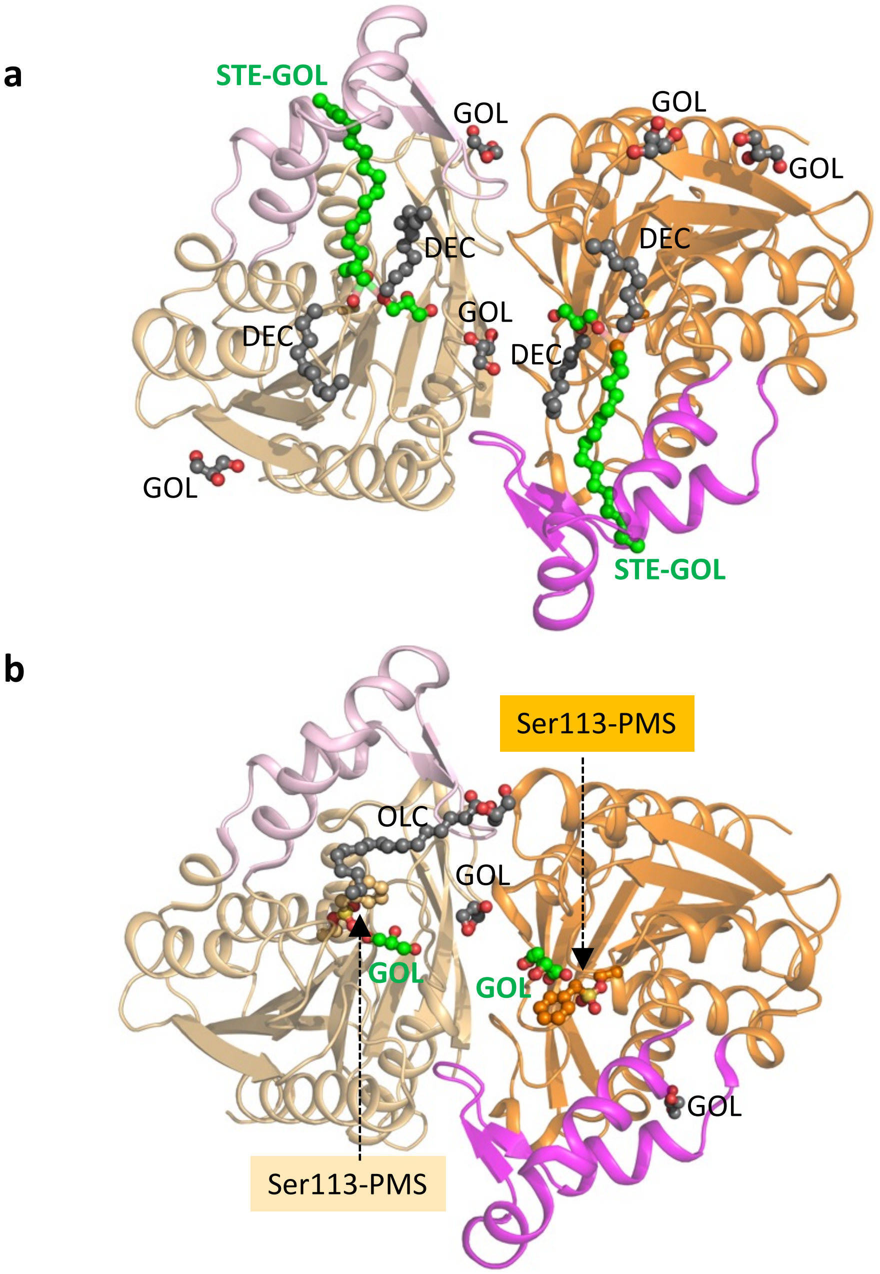

Supplementary Figure 14. Ligands found **a,** in the *Tth* MAG lipase Ser113-C18-MAG intermediate complex (PDB entry: 7Q4J) and **b,** in the *Tth* MAG lipase Ser113-PMS complex: STE-GOL, stearate-glycerol intermediate; DEC, alkane fragments tentatively assigned to contain 8,9 or 10 carbon atoms; OLC, 2,3-dihydroxypropyl-octadec-9-enoate; GOL, glycerol. Protein and validated ligand color codes are as in **Fig. 1** and **2**. Remaining, non-validated ligands are in grey and labeled.

Supplementary Tables

Supplementary Table 1: Thermostability of *Tth* variants

***Tth* lipase variant**	**Tm (°C)**	**ΔT_m(°C)**
wt	77.2 ± 0.16	
wt-PMS	77.1 ± 0.04	- 0.1
ΔLid	82.8 ± 0.01	+ 5.6
E43A	76.1 ± 0.02	- 1.1
E43K	77.9 ± 0.21	+ 0.7
E72R	75.7 ± 0.05	- 1.5
S113A	81.8 ± 0.07	+ 4.6
Y154A	78.3 ± 0.14	+ 1.1
Y154R	78.3 ± 0.10	+ 1.1

Legend Supplementary Table 1: We did not detect significant changes in thermostability (< 2 degrees changes), except for a variant without the lid domain (Δ140-183) that revealed an increased melting temperature of 83 °C, which is 6 °C higher than one found for the wt *Tth* MAG lipase **(Fig. 1b)**. This was within the melting temperature of the wt *Tth* MAG lipase **(Fig. 1b)**. This was within expectation as the lid domain is highly exposed and presents the most dynamic part of the overall *Tth* MAG lipase structure (**Fig. 1 & 2).** Interestingly, we have also observed a similar level of thermal stabilization in the *Tth* MAG lipase S113A active site mutant. The most plausible explanation is that a mutation of this active site residue could lead to a relieve of the high-energy ε-conformation of Ser113 found in both *wt* enzymes in the presence and absence of PMSF **(Supplementary Fig. 6)**. Data are presented as mean values ± SD, *n* = 3.

Supplementary Table 2: Data collection, phasing and refinement statistics

	Native	Active Se-Met (methylated)		PMS-inhibited (methylated) derivative
**Data collection**				
Space group	$P3_2$	$P4_32_12$		$P4_322$
Cell dimensions				
a, *b*, *c* (Å)	125.2, 125.2, 101.4	137.5, 137.5, 67.8		85.8, 85.8, 157.5
α, β, γ (°)	90, 90, 120	90, 90, 90		90, 90, 90
		Peak	*Inflection*	
Wavelength	0.9763	0.97873	0.97903	0.95372
Resolution (Å)	20.0 -2.42	50.0-2.56	20.0-1.91	30.0-2.00
	(2.46-2.42)	(2.60-2.56)	(1.94-1.91)	(2.05-2.00)
R_{sym} (%)	9.1 (61.9)	5.9 (13.1)	5.1 (64.2)	8.2 (51.4)
$I / \sigma I$	14.8 (2.7)	35.3 (19.3)	21.4 (2.03)	24.0 (1.65)
Completeness (%)	99.9 (99.1)	99.7 (100.0)	97.1 (82.2)	98.1 (82.2)
Redundancy	4.2 (3.3)	10.4 (10.0)	3.3 (2.9)	5.1 (2.7)
**Refinement**				
Resolution (Å)	19.94-2.42		19.75-1.91	29.98-2.00
No. reflections	65,618		49,195	38,285
R_{work} / R_{free} (%)	21.82/25.47		16.40/19.39	18.72/24.72
No. atoms				
Protein	12,246		4,135	4,101
Ligand/ion	66		136	65
Water	340		287	136
B-factors				
Protein	59.32		44.37	48.85
Ligand/ion	51.41		62.68	67.06
Water	51.40		48.98	44.22
R.m.s deviations				
Bond lengths (Å)	0.007		0.004	0.017
Bond angles (°)	1.026		0.833	1.894

*Values in parentheses are for highest-resolution shell. For positioning of ligands, see **Supplementary Fig. 14.**

Supplementary Table 3*: In vitro* activity data

Figure 4b, left panel: *Tth* MAG lipase (wt) activity towards MAG, DAG, TAG substrates [U mg^{-1}]

MAG (C8)	**DAG (C8)**	**TAG (C8)**	**MAG (C18)**
16.40 ± 1.30	0.23 ± 0.11	0.00 ± 0.30	0.79 ± 0.53

Figure 4b, right panel: *Tth* MAG lipase variant activity towards MAG substrate [U mg^{-1}]

	**C8**
wt	17.99 ± 1.00
E43A	5.97 ± 0.14
E43K	0.00 ± 0.27
E72R	0.00 ± 0.25
Y154A	13.49 ± 0.72
Y154R	5.32 ± 0.44
S113A	0.62 ± 0.28
ΔLid	0.13 ± 0.29

Figure 4c: *Tth* MAG lipase (wt) activity towards *p*NP substrate [U mg^{-1}]

C2	**C4**	**C5**	**C6**	**C8**	**C10**
1.23 ± 0.06	3.46 ± 0.15	3.56 ± 0.24	2.77 ± 0.18	2.49 ± 0.16	1.67 ± 0.13
C12	C14	C16	C18	ferulate	
0.98 ± 0.05	0.55 ± 0.04	0.04 ± 0.01	0.01 ± 0.01	0.03 ± 0.03	

Supplementary Table 4: Quantification of CD data

	Helix	Sheet	Turn	Sum	Other	RMSD[a)]	NRMSD[a)]
**wt**	36.5	18.1	17.6	72.2	27.7	7.9	2.9
**wt (PMSF)**	35.5	18.9	17.4	71.8	28.2	8.2	3.1
**E43A**	36.5	18.1	17.6	72.2	27.7	7.9	2.9
**E43K**	35.5	18.9	17.4	71.8	28.2	8.2	3.1
**E72R**	37.2	17.6	17.0	71.8	28.2	7.3	2.6
**S113A**	36.6	18.1	17.5	72.2	27.8	8.3	3.1
**Δ(lid)**	38.2	16.9	17.0	72.1	28.0	9.8	3.5
**Y154A**	38.4	17.1	16.8	72.3	27.7	7.0	2.5
**Y154R**	39.2	16.5	16.8	72.5	27.5	7.3	2.5

[a)] Root mean squares deviation and normalized root mean squares deviation as defined in CONTIN [3].

Supplementary Table 5: Water solubility of *p*-nitrophenyl FA esters used for *Tth* MAG lipase activity measurements

**Name**	**Corresponding acid**	**Water solubility (mg ml^{-1})[1]**
4-Nitrophenyl acetate (*p*NP-C2)	Acetic/ethanoic acid	1160
4-Nitrophenyl butyrate (*p*NP-C4)	Butyric/Butanoic acid	24
4-Nitrophenyl valerate (*p*NP-C5)	Valeric/Pentanoic acid	9
4-Nitrophenyl hexanoate (*p*NP-C6)	Caproic/Hexanoic acid	15
4-Nitrophenyl octanoate (*p*NP-C8)	Caprylic/Octanoic acid	1.513
4-Nitrophenyl decanoate (*p*NP-C10)	Capric/Decanoic acid	0.1515
4-Nitrophenyl laurate (*p*NP-C12)	Lauric/Dodecanoic acid	0.01504
4-Nitrophenyl myristate (*p*NP-C14)	Myristic/Tetradecanoic acid	0.001481
4-Nitrophenyl palmitate (*p*NP-C16)	Palmitic/Hexadecanoic acid	0.0001449
4-Nitrophenyl stearate (*p*NP-C18)	Stearic/Octadecanoic acid	0.00001409

Supplementary Table 6: Steady-state kinetics of *Tth* MAG lipase variants with *p*-nitrophenyl substrates of different chain lengths

***p*NP-C2**	K_M [mM]	k_{cat} [s^{-1}]	k_{cat}/K_M [M^{-1} s^{-1}]	$(k_{cat}/K_M)_{variant/wt}$
Wt	2.3 ± 0.3	5.2 ± 0.6	2.2 ± 0.4 x 10^3	1.0
E43A	1.9 ± 0.3	1.6 ± 0.2 x 10^1	8.3 ± 1.4 x 10^3	3.7
E43K	6.3 ± 0.6 x 10^{-1}	1.0 ± 0.0	1.5 ± 0.2 x 10^3	0.7
E72R	7.1 ± 0.7 x 10^{-1}	4.0 ± 0.2	5.7 ± 0.6 x 10^3	2.5
Y154A	1.4 ± 1.1 x 10^{-1}	2.1 ± 1.5 x 10^1	1.5 ± 1.6 x 10^3	0.7
Y154R	2.0 ± 0.3	1.3 ± 0.1 x 10^1	6.3 ± 1.2 x 10^3	2.8

***p*NP-C4**	K_M [mM]	k_{cat} [s^{-1}]	k_{cat}/K_M [M^{-1} s^{-1}]	$(k_{cat}/K_M)_{variant/wt}$
wt	7.1 ± 0.7 x 10^{-1}	8.6 ± 0.5	1.2 ± 0.1 x 10^4	1.0
E43A	6.7 ± 1.9 x 10^{-1}	1.6 ± 0.2 x 10^1	2.4 ± 0.8 x 10^4	2.0
E43K	8.2 ± 0.8 x 10^{-1}	4.4 ± 0.2	5.3 ± 0.6 x 10^3	0.4
E72R	1.4 ± 2.0	5.6 ± 5.2	3.9 ± 6.5 x 10^3	0.3
Y154A	3.4 ± 0.6	1.3 ± 0.2 x 10^1	3.8 ± 0.9 x 10^3	0.3
Y154R	7.2 ± 1.9 x 10^{-1}	1.2 ± 0.2 x 10^1	1.6 ± 0.5 x 10^4	1.3

***p*NP-C5**	K_M [mM]	k_{cat} [s^{-1}]	k_{cat}/K_M [M^{-1} s^{-1}]	$(k_{cat}/K_M)_{variant/wt}$
wt	8.3 ± 1.3 x 10^{-1}	8.4 ± 0.7	1.0 ± 0.2 x 10^4	1.0
E43A	6.5 ± 1.6 x 10^{-1}	1.5 ± 0.2 x 10^1	2.2 ± 0.6 x 10^4	2.2
E43K	4.5 ± 0.8 x 10^{-1}	3.2 ± 0.3	7.3 ± 1.5 x 10^3	0.7
E72R	9.4 ± 3.3 x 10^{-1}	1.1 ± 0.2 x 10^1	1.2 ± 0.5 x 10^4	1.2
Y154A	1.4 ± 0.2	1.1 ± 0.1 x 10^1	8.1 ± 1.6 x 10^3	0.8
Y154R	8.8 ± 2.2 x 10^{-1}	1.3 ± 0.2 x 10^1	1.5 ± 0.4 x 10^4	1.5

***p*NP-C6**	K_M [mM]	k_{cat} [s^{-1}]	k_{cat}/K_M [M^{-1} s^{-1}]	$(k_{cat}/K_M)_{variant/wt}$
wt	3.6 ± 0.8 x 10^{-1}	4.9 ± 0.4	1.4 ± 0.3 x 10^4	1.0
E43A	7.1 ± 3.0 x 10^{-1}	1.2 ± 0.3 x 10^1	1.8 ± 0.8 x 10^4	1.3
E43K	3.2 ± 0.9 x 10^{-1}	2.8 ± 0.3	8.7 ± 2.7 x 10^3	0.6
E72R	6.3 ± 3.4 x 10^{-1}	8.3 ± 2.3	1.3 ± 0.8 x 10^4	1.0
Y154A	4.8 ± 0.8 x 10^{-1}	5.7 ± 0.4	1.2 ± 0.2 x 10^4	0.9
Y154R	7.5 ± 3.6 x 10^{-1}	1.2 ± 0.3 x 10^1	1.5 ± 0.8 x 10^4	1.1

***p*NP-C8**	K_M [mM]	k_{cat} [s^{-1}]	k_{cat}/K_M [M^{-1} s^{-1}]	$(k_{cat}/K_M)_{variant}/_{wt}$
wt	$4.9 \pm 1.5 \times 10^{-1}$	5.5 ± 0.8	$1.1 \pm 0.4 \times 10^{4}$	1.0
E43A	$5.2 \pm 3.4 \times 10^{-1}$	$1.1 \pm 0.3 \times 10^{1}$	$2.2 \pm 1.6 \times 10^{4}$	1.9
E43K	$4.6 \pm 1.3 \times 10^{-1}$	3.5 ± 0.5	$7.7 \pm 2.4 \times 10^{3}$	0.7
E72R	$5.1 \pm 2.2 \times 10^{-1}$	6.4 ± 1.3	$1.3 \pm 0.6 \times 10^{4}$	1.1
Y154A	$6.2 \pm 2.3 \times 10^{-1}$	6.4 ± 1.2	$1.0 \pm 0.4 \times 10^{4}$	0.9
Y154R	$8.2 \pm 4.4 \times 10^{-1}$	$1.1 \pm 0.3 \times 10^{1}$	$1.3 \pm 0.8 \times 10^{4}$	1.2

***p*NP-C10**	K_M [mM]	k_{cat} [s^{-1}]	k_{cat}/K_M [M^{-1} s^{-1}]	$(k_{cat}/K_M)_{variant}/_{wt}$
wt	$3.0 \pm 0.7 \times 10^{-1}$	4.4 ± 0.4	$1.5 \pm 0.4 \times 10^{4}$	1.0
E43A	$2.7 \pm 1.0 \times 10^{-1}$	7.2 ± 1.0	$2.6 \pm 1.0 \times 10^{4}$	1.8
E43K	$3.3 \pm 1.1 \times 10^{-1}$	2.8 ± 0.4	$8.6 \pm 2.9 \times 10^{3}$	0.6
E72R	$1.3 \pm 0.7 \times 10^{-1}$	2.4 ± 0.4	$1.9 \pm 1.1 \times 10^{4}$	1.3
Y154A	$2.2 \pm 0.6 \times 10^{-1}$	2.9 ± 0.3	$1.3 \pm 0.4 \times 10^{4}$	0.9
Y154R	$3.9 \pm 1.1 \times 10^{-1}$	7.7 ± 0.9	$2.0 \pm 0.6 \times 10^{4}$	1.3

***p*NP-C12**	K_M [mM]	k_{cat} [s^{-1}]	k_{cat}/K_M [M^{-1} s^{-1}]	$(k_{cat}/K_M)_{variant}/_{wt}$
wt	$1.1 \pm 0.1 \times 10^{-1}$	1.9 ± 0.1	$1.8 \pm 0.2 \times 10^{4}$	1.0
E43A	$1.0 \pm 0.1 \times 10^{-1}$	3.2 ± 0.1	$3.3 \pm 0.5 \times 10^{4}$	1.8
E43K	$5.3 \pm 1.3 \times 10^{-2}$	$9.4 \pm 0.6 \times 10^{-1}$	$1.8 \pm 0.5 \times 10^{4}$	1.0
E72R	$1.7 \pm 0.6 \times 10^{-2}$	$4.0 \pm 0.3 \times 10^{-1}$	$2.4 \pm 0.9 \times 10^{4}$	1.3
Y154A	$6.4 \pm 0.9 \times 10^{-2}$	1.1 ± 0.0	$1.7 \pm 0.2 \times 10^{4}$	0.9
Y154R	$1.2 \pm 0.2 \times 10^{-1}$	2.8 ± 0.1	$2.4 \pm 0.3 \times 10^{4}$	1.3

***p*NP-C14**	K_M [mM]	k_{cat} [s^{-1}]	k_{cat}/K_M [M^{-1} s^{-1}]	$(k_{cat}/K_M)_{variant}/_{wt}$
wt	$1.2 \pm 0.1 \times 10^{-1}$	$5.0 \pm 0.1 \times 10^{-1}$	$4.2 \pm 0.4 \times 10^{3}$	1.0
E43A	$1.0 \pm 0.1 \times 10^{-1}$	$8.3 \pm 0.2 \times 10^{-1}$	$8.2 \pm 0.8 \times 10^{3}$	1.9
E43K	$4.3 \pm 1.2 \times 10^{-2}$	$1.7 \pm 0.1 \times 10^{-1}$	$3.9 \pm 1.1 \times 10^{3}$	0.9
E72R	$4.4 \pm 1.4 \times 10^{-2}$	$3.4 \pm 0.3 \times 10^{-1}$	$7.7 \pm 2.6 \times 10^{3}$	1.8
Y154A	$9.0 \pm 1.3 \times 10^{-2}$	$3.8 \pm 0.2 \times 10^{-1}$	$4.2 \pm 0.6 \times 10^{3}$	1.0
Y154R	$1.7 \pm 0.7 \times 10^{-1}$	1.0 ± 0.1	$6.1 \pm 2.6 \times 10^{3}$	1.5

Supplementary Table 7: FAME data

Normalized activity (ratio variant *Tth* lipase/ wt *Tth* lipase)

	wt	∆Lid	S113A	E43A	E43K	Y154A	Y154R
	1.080	0.616	0.680	2.148	0.962	0.983	1.595
	0.992	0.894	0.704	2.091	0.902	0.915	1.686
	1.084	0.840	0.437	1.769	0.614	1.059	0.756
	1.046	0.498			0.618	0.894	
	0.918	0.952					
	0.879	0.644					
		0.447					
No. experiments	n=6	n=7	n=3	n=3	n=4	n=4	n=3

Statistical analysis: unpaired t-test; two-tailed p values (GraphPad Prism 5.04)

	wt	∆Lid	S113A	E43A	E43K	Y154A	Y154R
Mean	1.000	0.699	0.607	2.003	0.774	0.963	1.346
SD	0.086	0.198	0.148	0.204	0.184	0.075	0.513
p value *vs*. wt		0.0055	0.0013	< 0.0001	0.0288	0.4985	0.1285
p value summary		**	**	***	*		

Supplementary Table 8: *Tth* MAG lipase sequence/structure relations to other α/β hydrolases

Sequence ID	Protein name	Organism	Phylum	Reference
I9KU82	MAG lipase	*T. thermohydrosulfuricus*	Firmicutes	
D3YEX6	Cinnamoyl esterase	*L. johnsonii*	Firmicutes	2
D2YW37	Feruloyl esterase	*B. proteoclasticus*	Firmicutes	1
D0MHY8	Osmotically inducible protein C	*R. marinus*	Bacteroidetes	4
B9JYM4	Hydroxymuconic semialdehyde hydrolase	*A. vitis*	Proteobacteria	unpublished
Q6PE15	Palmitoyl-protein thioesterase A	*M. musculus*	(Vertrebrate)	5
Q83AV9	Alpha/beta hydrolase	*C. burnetii*	Proteobacteria	6
O30361	Xylan esterase	*T. saccharolyticum*	Firmicutes	unpublished
Q65EQ1	Carboxylesterase	*B. licheniformis*	Firmicutes	unpublished
Q1QEU6	Triacylglycerol lipase	*P. cryohalolentis*	Proteobacteria	unpublished
A0A6I8WFT7	MAG lipase	*P. ferrophilus*	Euryarchaeota	unpublished
B0V9K7	Dienelactone hydrolase	*A. baumannii*	Proteobacteria	unpublished
A0A6L8NUA5	Bromoperoxidase	*B. anthracis*	Firmicutes	unpublished
P82597	MAG lipase	*Bacillus sp.*	Firmicutes	7
O07427	MAG lipase	*M. tuberculosis*	Actinobacteria	8
A0A0B5WSQ6	Lysophospho- lipase	*B. coagulans*	Firmicutes	9
J4WAT9	AB hydrolase-1	*B. bassiana*	Ascomycota	10

Sequence ID	Score[a]	E value[a]	PDB ID[b,c]	Q score[b]	Rmsd[b]	Seq. identity[b]	Lid[d] type	Lid length[d]	Assembly
I9KU82			7Q4J				HBH	43	Dimer
D3YEX6	117.0	1.00E-31	3S2Z*	0.72	1.18	0.32	HBH	46	Dimer
D2YW37	102.0	1.00E-25	2WTM*	0.68	1.31	0.30	HBH	46	Dimer
D0MHY8	82.0	2.00E-17	5CML	0.44	2.22	0.23	HBH**	48	Dimer
B9JYM4	55.8	9.00E-09	3LLC	0.56	1.88	0.22	HBH	50	Monomer
Q6PE15	51.6	3.00E-07	6NY9	0.52	1.99	0.20	HBH	51	Monomer
Q83AV9	47.4	4.00E-06	*3TRD*				*no*		*Monomer*

O30361	39.3	0.003	3FCY	0.32	2.11	0.20	HB	43	Trimer
Q65EQ1	38.5	0.005	6NKG				HB	54	Monomer
Q1QEU6	37.4	0.014	4NS4	0.36	2.03	0.22	HB	54	Monomer
A0A6I8WFT7	34.7	0.1	6QE2				HBH	66	Dimer
B0V9K7	34.7	0.089	4OPM				HB	70	Dimer
A0A6L8NUA5	33.5	0.23	3FOB				HB	80	Trimer
P82597	32.7	0.41	4KE8*	0.45	2.16	0.21	HBH	51	Dimer
O07427	30.4	2.30	6EIC	0.45	1.86	0.18	HBH	70	Trimer
A0A0B5WSQ6	28.9	7.7	5O7G*	0.39	2.13	0.15	HB	88	Monomer
J4WAT9			7D79*	0.49	2.00	0.19	HBH	84	Dimer

A BLAST search using the *Tth* MAG lipase sequence (A0A1I1X7Z7) against UniProtKB restricted to sequences with known 3D structures revealed 106 hits. From this pool of sequences, 15 non-redundant validated targets were selected **(Supplementary Fig. 12)**. These sequences were further analyzed by structural superposition using the PDBeFold server [11]. For ten out of fifteen selected hits, PDBeFold produced a reliable structure-based alignment for both catalytic domain segments (**Supplementary Fig. 5**). In addition, an α/β hydrolase from *Beauveria bassiana* (J4WAT9) was included, which was detected in the PDBeFold search but not in the BLAST search **(Supplementary Data 1)**.

[a] Taken from **Supplementary Data 1.**

[b] Taken from PDBeFold output using the structure of *Tth* MAG lipase as template against the PDB.

[c], *, indicates PDB code cited represents multiple structures deposited in PDB of the same sequence.

[d], for further details see **Fig. 7a**; **, indicates a non-canonical HBH lid domain (for further details, see text)

Supplementary Table 9: Primers used for mutagenesis

**E43A**
Forward: 5′-GGTTTTACAGGCAATAAAGTA**GCG**TCTCACTTTATTTTTGTGAAG-3′<br>Reverse: 5′-CTTCACAAAAATAAAGTGAGACGCTACTTTATTGCCTGTAAAACC-3′
**E43K**
Forward: 5′-GGTTTTACAGGCAATAAAGTA**AAG**TCTCACTTTATTTTTGTGAAG-3′<br>Reverse: 5′-CTTCACAAAAATAAAGTGAGACTTTACTTTATTGCCTGTAAAACC-3′
**E72R**
Forward: 5′-GACTTTTATGGTTCTGGA**AGA**AGTGATGGGGACTTTAGTGAA-3′<br>Reverse: 5′-TTCACTAAAGTCCCCATCACTTCTTCCAGAACCATAAAAGTC-3′
**S113A**
Forward: 5'-AGAATAGGACTACTTGGTTTG**GCC**ATGGGAGGAGCTATTGCAGGG-3'<br>Reverse: 5'-CCCTGCAATAGCTCCTCCCATGGCCAAACCAAGTAGTCCTATTCT-3'
**Y154R**
Forward: 5′-AACGAAAGTGTAAAGCAA**CGC**GGAGCTATTATGGAA-3′<br>Reverse: 5′-TTCCATAATAGCTCCGCGTTGCTTTACACTTTCGTT-3′
**Y154A**
Forward: 5′-AACGAAAGTGTAAAGCAA**GCC**GGAGCTATTATGGAA-3′<br>Reverse: 5′-TTCCATAATAGCTCCGGCTTGCTTTACACTTTCGTT-3′
**Lid deletion mutant (Δ140-183)**
Forward: 5′-AAAAGGTACCTTTGAGCTGTCAAAAGGATACGAT-3′<br>Reverse: 5′-AAAAGGTACCAAAAGCTGGAGCCCATAGCACCA-3′
**Subcloning from pETBlue-1 to pETM-14 for WT and mutants (SLiCe cloning)**
Forward: 5′-CTGGAAGTTCTGTTCCAGGGGCCCATGCAAAAGGCTGTTGAAATT – 3′<br>Reverse: 5′-CTTGTCGACGGAGCTCGAATTCGGCTATCCCTTTAACAATTCCTT-3′

Supplementary Table 10: *Tth* MAG lipase structure lysine methylation sites

Residue	7Q4J(A)	7Q4J(B)		7Q4H(A)	7Q4H(B)
**3**		D			D
**12**	D			M	
**25**		D			M
**27**					M
**41**					
**50**					
**57**		D			
**94**	D	M			
**97**	D				
**127**	D	D			
**131**		M			D
**152**		M			
**170**	D	D			
**173**					
**181**					
**189**				M	
**193**		D		D	
**194**	M	M			D
**209**	M	D			
**216**					
**236**	D	D		D	M
**242**	M				
**243**	D	D			
**253**	M	M		M	D
**254**		M			
**258**					
**Methyl groups (total)**	**18**	**24**		**7**	**11**

M, mono-methylated; D, di-methylated.

Supplementary Table 11: Geometry of C18 MAG-Ser113 intermediate (7Q4J)

***Tth* MAG lipase chains**	**A**	**B**
Distances [Å]		
C1(STE)-C2(STE)	1.53	1.56
C1(STE)-O1(STE)	1.33	1.34
C1(STE)-OG(Ser113)	2.18	2.30
C1(STE)-O1(GOL)	2.60	2.59
Angles [degrees]		
C2(STE)-C1(STE)-O1(STE)	110.9	112.6
C2(STE)-C1(STE)-OG(Ser113)	140.0	112.3
C2(STE)-C1(STE)-O1(GOL)	142.9	172.3
O1(STE)-C1(STE)-OG(Ser113)	95.3	89.1
O1(STE)-C1(STE)-O1(GOL)	71.7	74.6
OG(Ser113)-C1(STE)-O1(GOL)	73.1	69.4

Atom definitions are taken from PDB entry 7Q4J.

Supplementary References

1. Goldstone, D.C. et al. Structural and functional characterization of a promiscuous feruloyl esterase (Est1E) from the rumen bacterium Butyrivibrio proteoclasticus. *Proteins* **78**, 1457-69 (2010).

2. Lai, K.K. et al. An inserted α/β subdomain shapes the catalytic pocket of Lactobacillus johnsonii cinnamoyl esterase. *PLoS One* **6**, e23269 (2011).

3. Provencher, S.W. & Glöckner, J. Estimation of globular protein secondary structure from circular dichroism. *Biochemistry* **20**, 33-7 (1981).

4. Jensen, M.V. et al. Characterisation of a New Family of Carboxyl Esterases with an OsmC Domain. *PLoS One* **11**, e0166128 (2016).

5. Cao, Y. et al. ABHD10 is an S-depalmitoylase affecting redox homeostasis through peroxiredoxin-5. *Nat Chem Biol* **15**, 1232-1240 (2019).

6. Franklin, M.C. et al. Structural genomics for drug design against the pathogen Coxiella burnetii. *Proteins* **83**, 2124-36 (2015).

7. Rengachari, S. et al. Conformational plasticity and ligand binding of bacterial monoacylglycerol lipase. *J Biol Chem* **288**, 31093-104 (2013).

8. Aschauer, P., Zimmermann, R., Breinbauer, R., Pavkov-Keller, T. & Oberer, M. The crystal structure of monoacylglycerol lipase from M. tuberculosis reveals the basis for specific inhibition. *Sci Rep* **8**, 8948 (2018).

9. De Vitis, V. et al. A stereospecific carboxyl esterase from Bacillus coagulans hosting nonlipase activity within a lipase-like fold. *Febs j* **285**, 903-914 (2018).

10. Gao, D.W. et al. A Polyketide Cyclase That Forms Medium-Ring Lactones. *J Am Chem Soc* **143**, 80-84 (2021).

11. Krissinel, E. & Henrick, K. Secondary-structure matching (SSM), a new tool for fast protein structure alignment in three dimensions. *Acta Crystallogr D Biol Crystallogr* **60**, 2256-68 (2004).

Supplementary Source Data

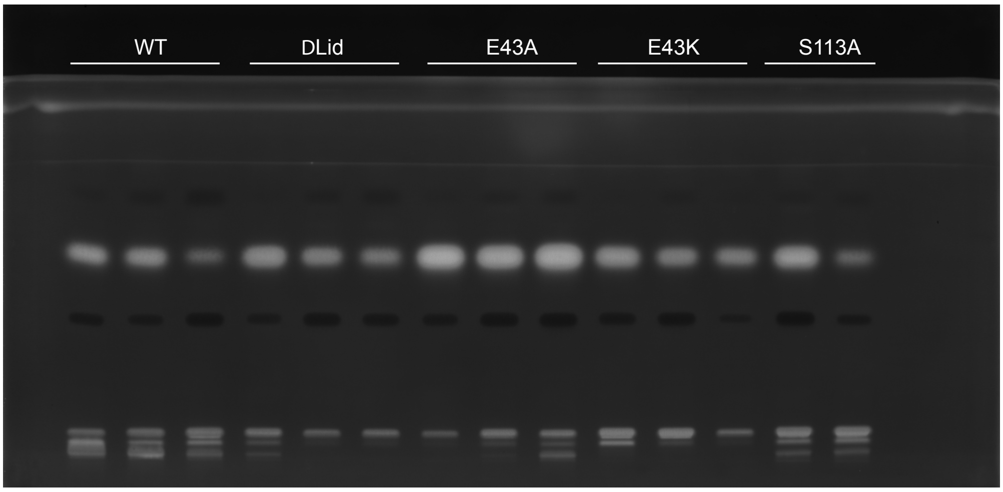

Uncropped scan of Figure 4f

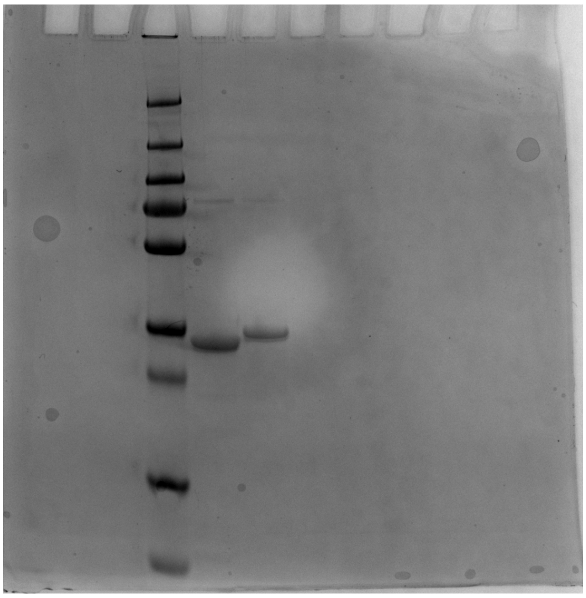

Uncropped scan of Supplementary Figure 13
