# Supplementary material for: Discovery of a non-canonical prototype long-chain monoacylglycerol lipase through a structure-based endogenous reaction intermediate complex
Source: Nat Commun. 2023 Nov 27;14:7649. doi: 10.1038/s41467-023-43354-4 (PMC10682391; doi:10.1038/s41467-023-43354-4)
Supplement: Supplementary file 3 — Description of Additional Supplementary Files [file 41467_2023_43354_MOESM3_ESM.pdf]

Description of Additional Supplementary Files

File Name: Supplementary Data 1
Description: A search against the UniProtKB50 protein sequence data base revealed the top scoring 1000 sequence families with a minimal threshold score of 70 and an E value of 7 x 10-
and archaeal phyla, to the extent organisms of origin were unambiguously determined.

File Name: Supplementary Data 2
Description: Source Data for Supplementary Figure 3.
