# Supplementary material for: Discovery of a non-canonical prototype long-chain monoacylglycerol lipase through a structure-based endogenous reaction intermediate complex
Source: Nat Commun. 2023 Nov 27;14:7649. doi: 10.1038/s41467-023-43354-4 (PMC10682391; doi:10.1038/s41467-023-43354-4)

measured by Richard Blaauw at Chiralix:
the major constituent having a mass of 359 using C.I. in positive ionization mode (corresponding to M.W. + 1) can be seen. Whether or not you want to use it in the publication is up to you.

Chiralix
innovative drug discovery solutions

Sample Information

Aquisition Date and Time	: 5/19/2008 18:18:53 PM
Sample Name	: N. Pinotsis (EMBL)
Injection Volume	: 20 µL
Column	: C18
Method	: AB Gradient 50%A - 95%A (0-5 min), 95%A (5-10 min)
Solvent A	: acetonitrile (0.1% TFA)
Solvent B	: water (0.1% TFA)

LC Chromatogram

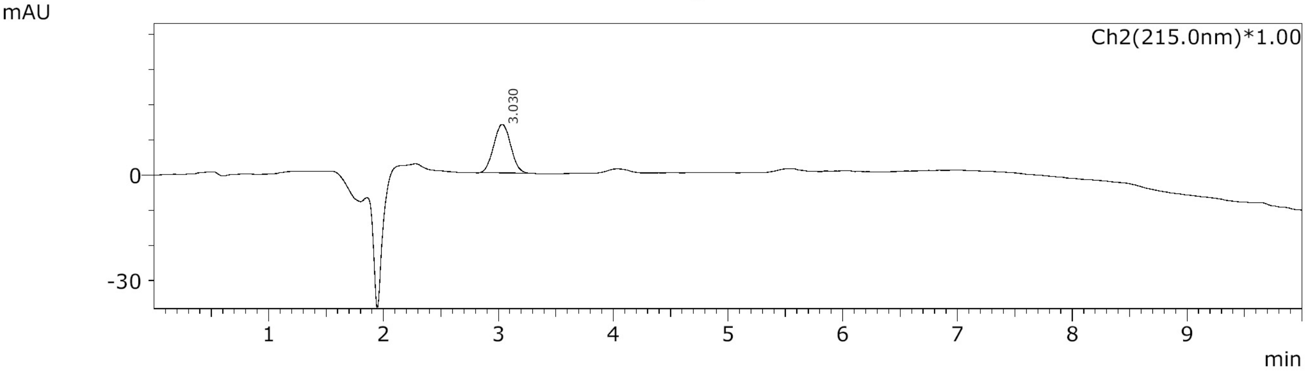

LC Peak Table - Ch2

Peak#	R.Time	Area	Area%
1	3.030	141872	100.00
		141872	100.00

MS Chromatogram

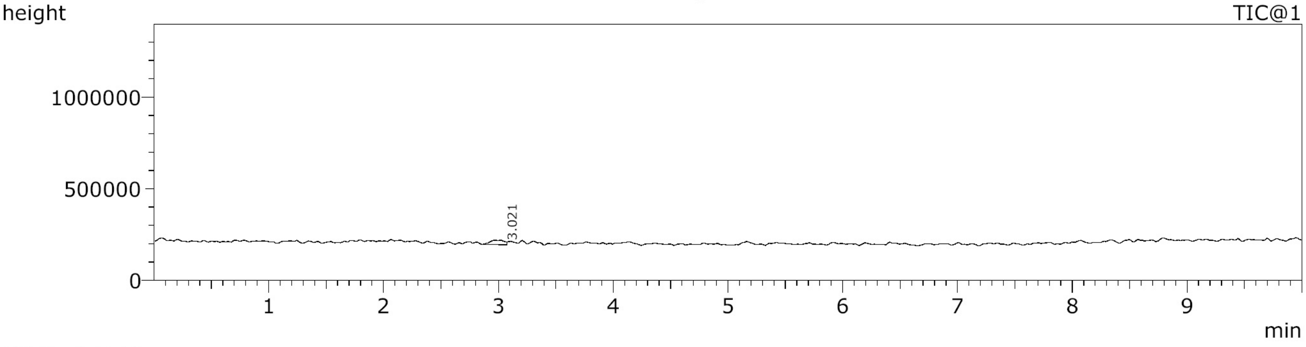

MS Peak Table

Peak#	R.Time	Base m/z
1	3.021	359.10

MS Spectrum

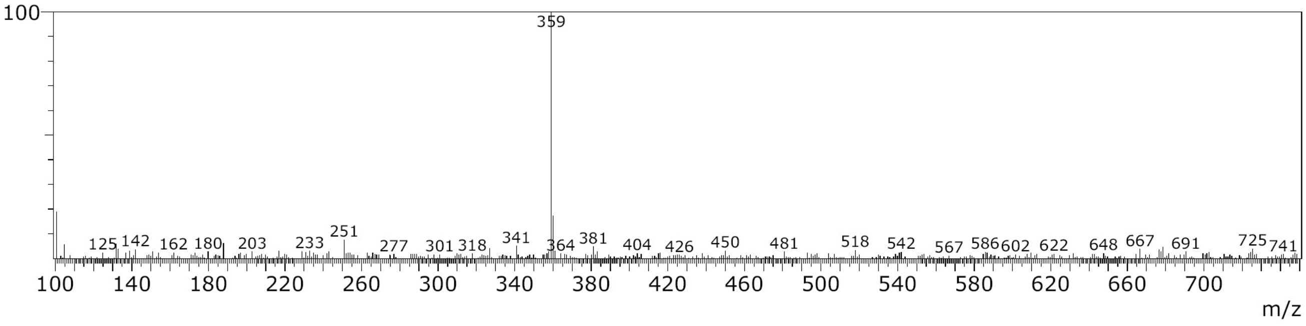

Supplement: Supplementary file 5 — Supplementary Data 2 [file 41467_2023_43354_MOESM5_ESM.zip › Supplement Figure3b.pdf]
