# Supplementary material for: Discovery of a non-canonical prototype long-chain monoacylglycerol lipase through a structure-based endogenous reaction intermediate complex
Source: Nat Commun. 2023 Nov 27;14:7649. doi: 10.1038/s41467-023-43354-4 (PMC10682391; doi:10.1038/s41467-023-43354-4)
Supplement: Supplementary file 5 — Supplementary Data 2 [file 41467_2023_43354_MOESM5_ESM.zip › SupplementFigure13b.pdf]

Intact mass measurement
P2309 Sample 1 non-methylated

Chromatogram

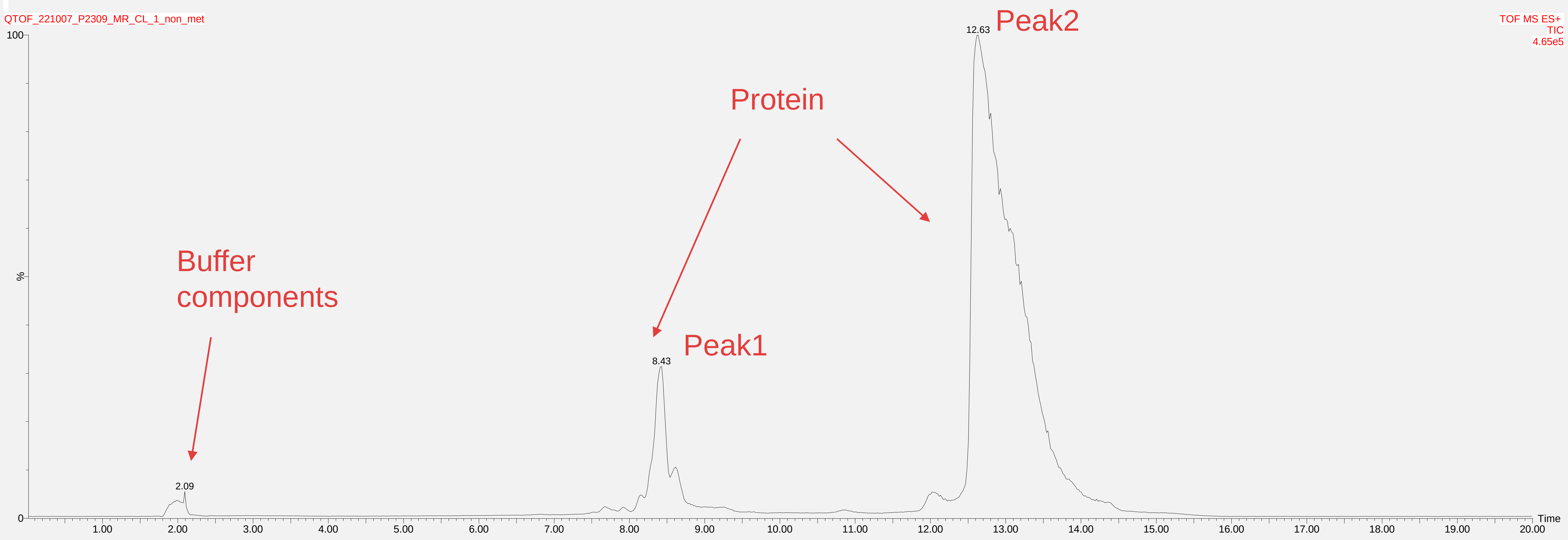

EMBL

Spectrum and Mass – Peak1

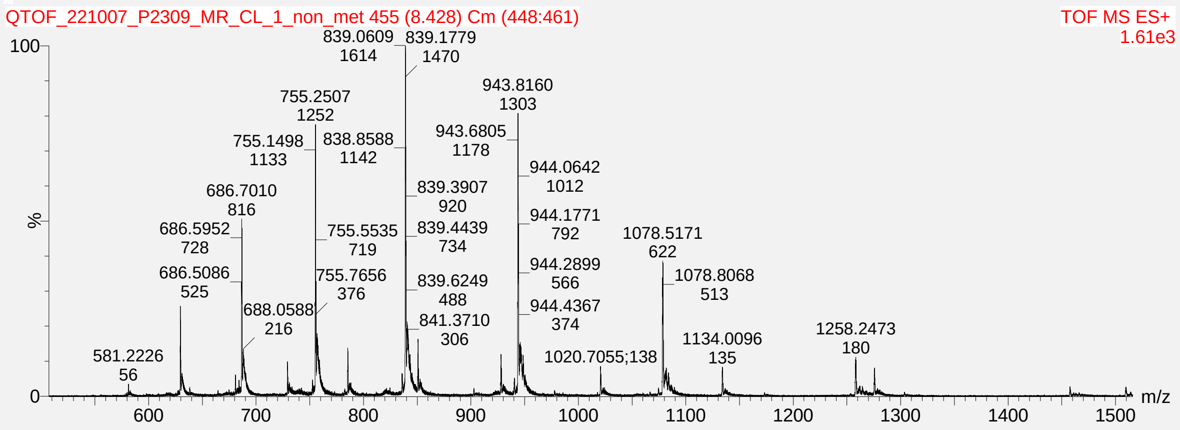

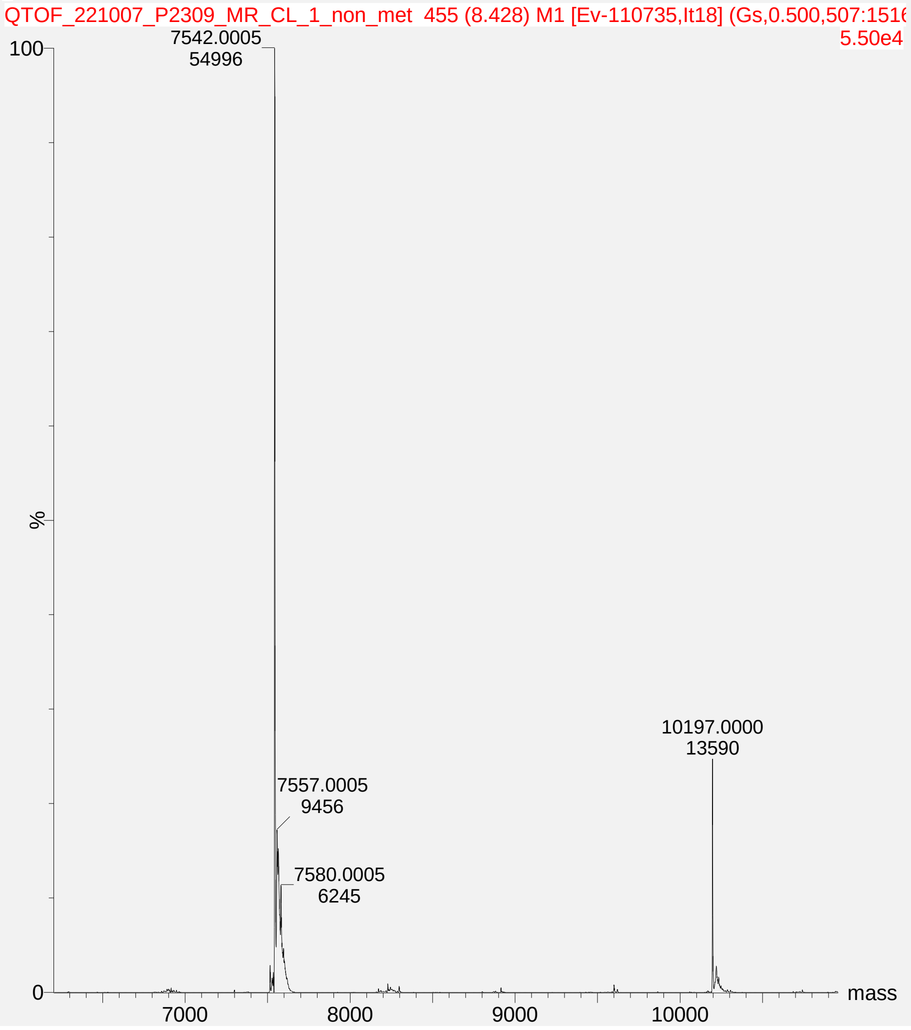

Measured Mass:

- 7542 Da
- 10197 Da

EMBL

Spectrum and Mass – Peak2

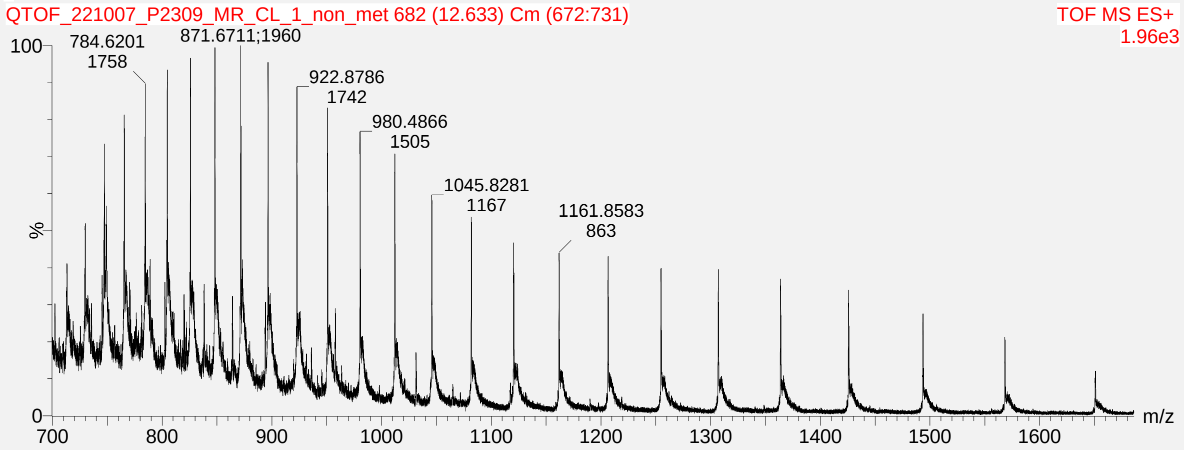

Expected Mass: 29163.54 Da
Measured Mass: 31345 Da (+2182Da)

Compute pI/Mw

Theoretical pI/Mw (average) for the user-entered sequence:

```
        10         20         30         40         50         60
MQKAVEITYN GKTLRGMMHL PDDVKGKVPM VIMFHGFTGN KVESHFIFVK MSRALEKVGI

        70         80         90        100        110        120
GSVRFDFYGS GESDGDFSEM TFSSELEDAR QILKFVKEQP TTDPERIGLL GLSMGGAIAG

       130        140        150        160        170        180
IVAREYKDEI KALVLWAPAF NMPELIMNES VKQYGAIMEQ LGFVDIGGHK LSKDFVEDIS

       190        200        210        220        230        240
KLNIFELSKG YDKKVLIVHG TNDEAVEYKV SDRILKEVYG DNATRVTIEN ADHTFKSLEW

       250
EKKAIEESVE FFKKELLKG
```

Theoretical pI/Mw: 5.40 / 29163.54

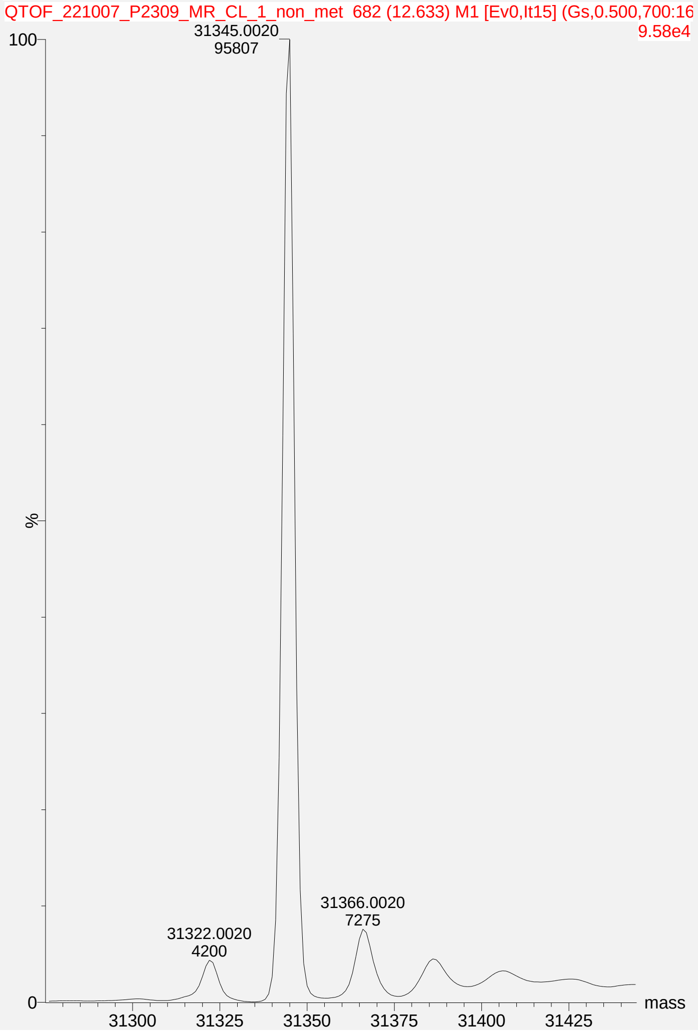

EMBL

Intact mass measurement
P2309 Sample 1 methylated

Chromatogram

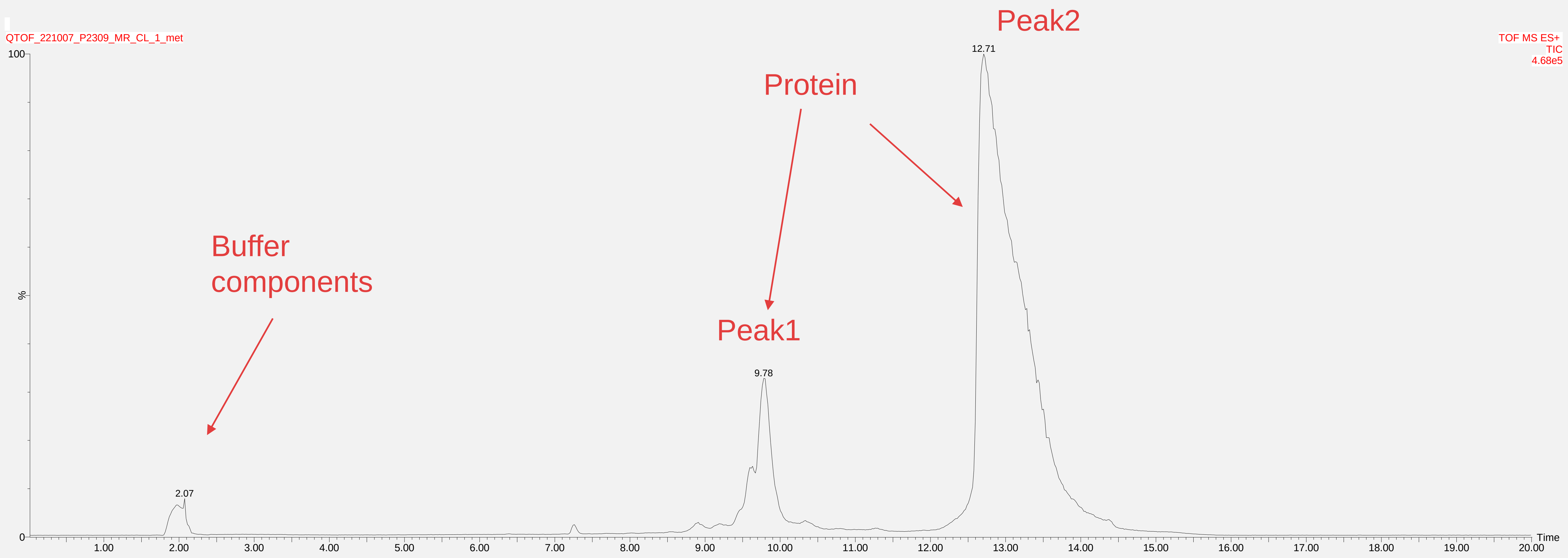

EMBL

Spectrum and Mass – Peak1

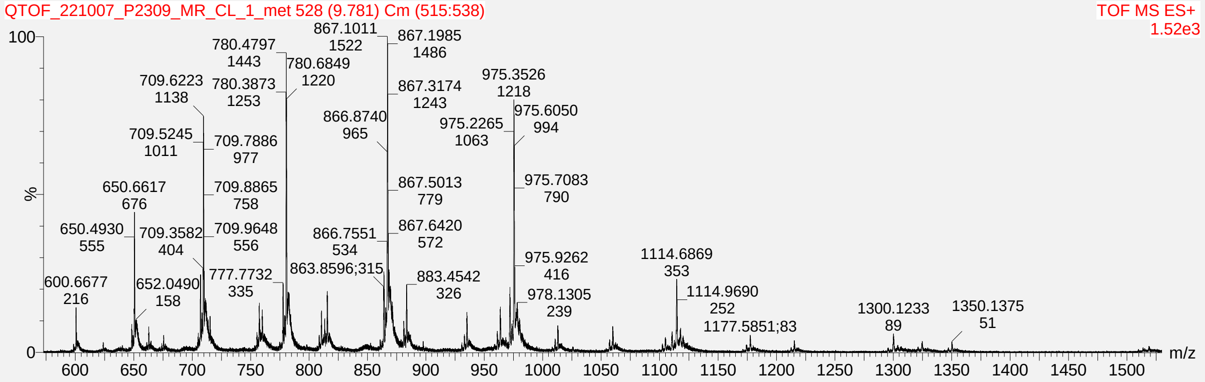

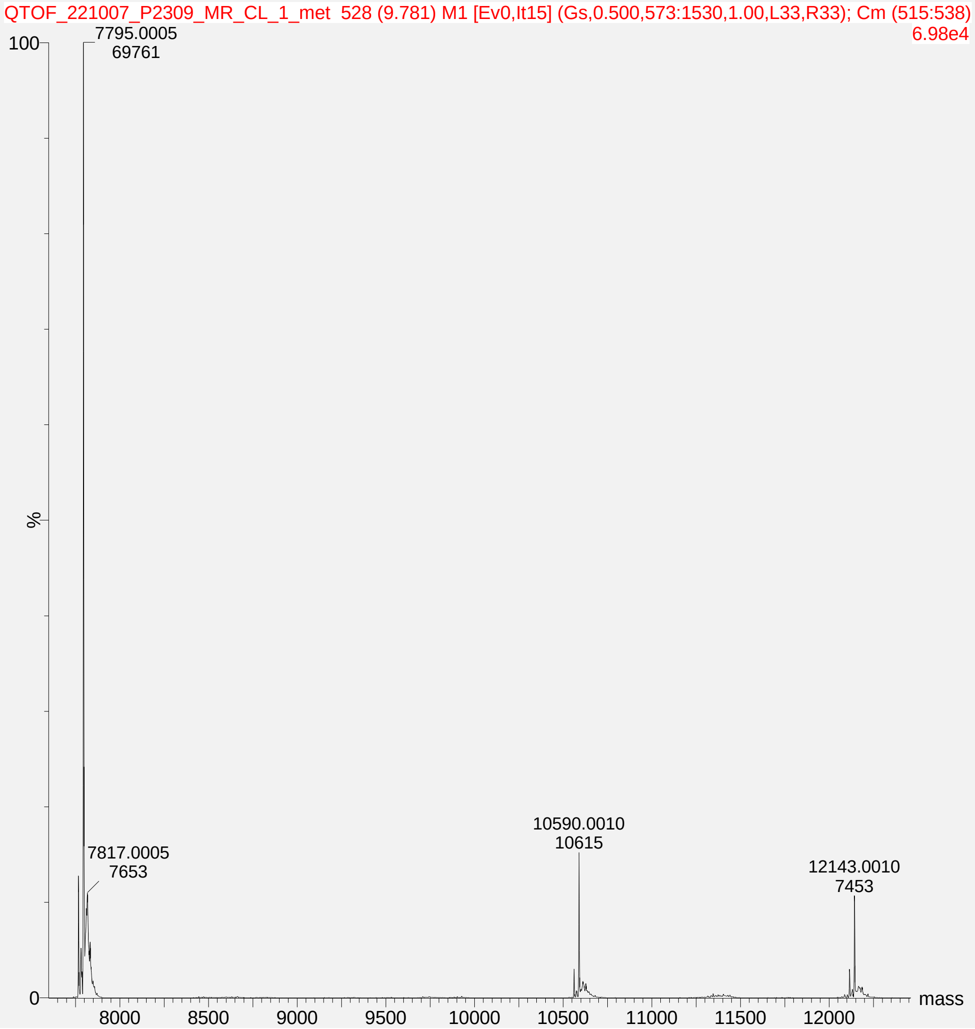

Measured Mass:

- 7795 Da
- 10590 Da
- 12143 Da

EMBL

Spectrum and Mass – Peak2

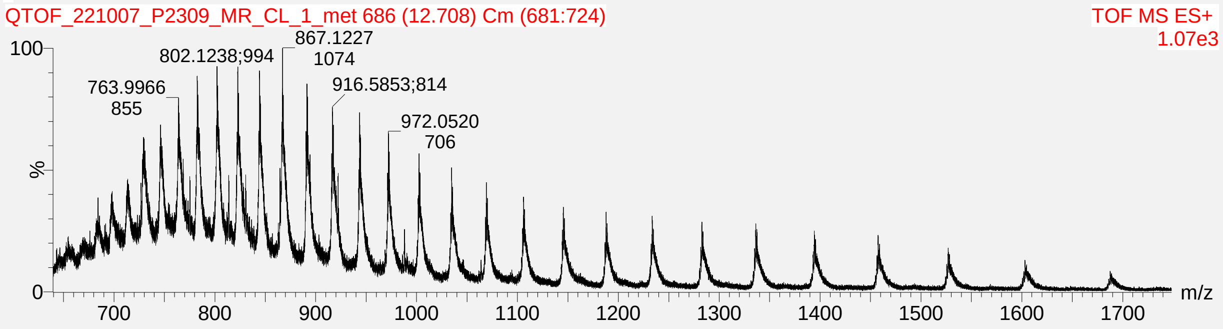

Expected Mass: 29163.54 Da
Measured Mass of non-modified: 31345 Da
Measured Mass:

- 32017 Da (+672 Da)
- 32046 Da (+701 Da)
- 32074 Da (+729 Da)

Difference: 29 Da

Difference: 28 Da

maybe a di-methylation pattern

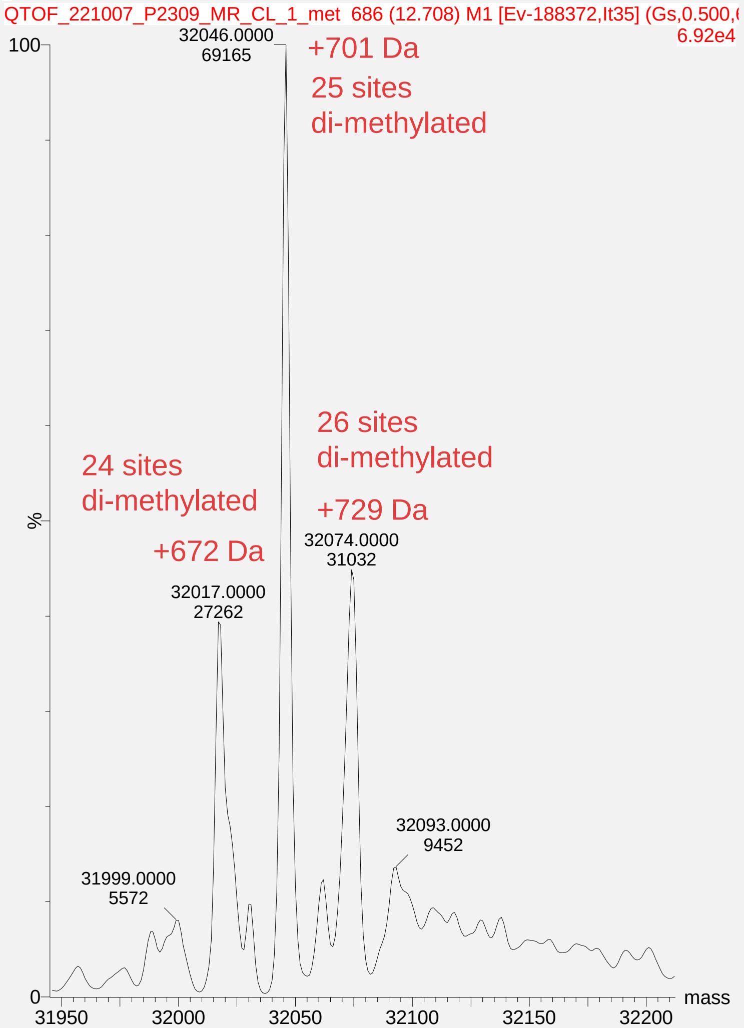

EMBL

Intact mass measurement
P2309 Sample 2 non-methylated

EMBL

Chromatogram

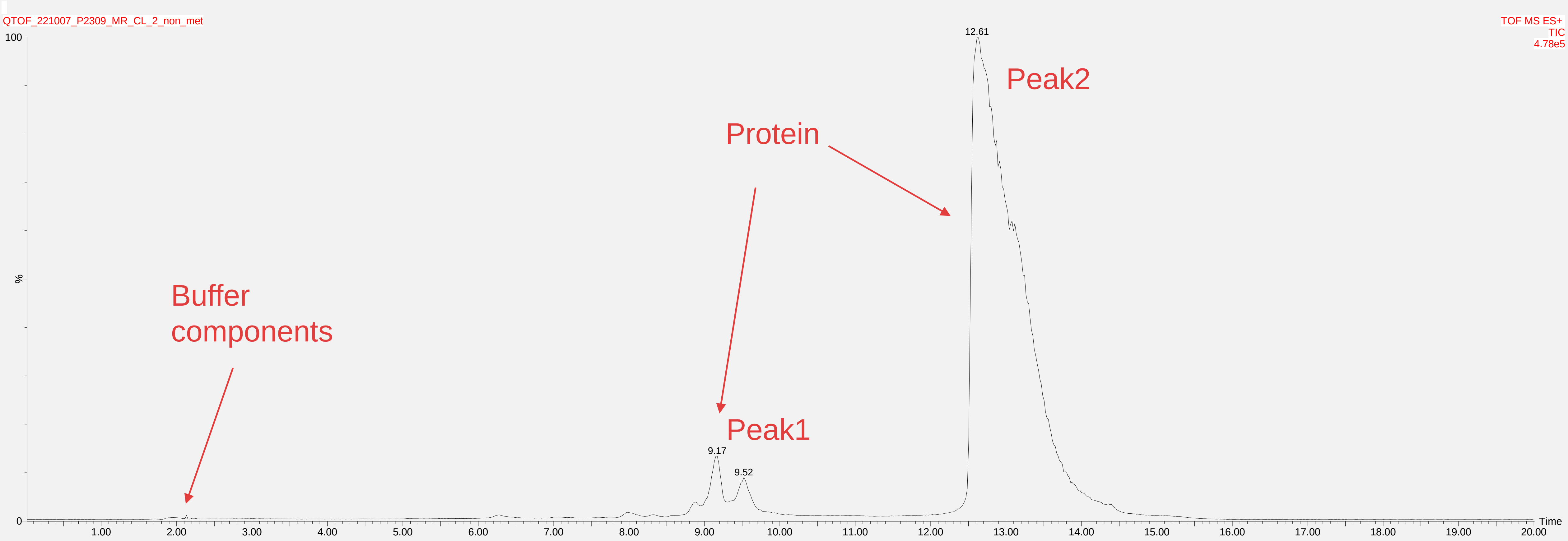

EMBL

Spectrum and Mass – Peak1

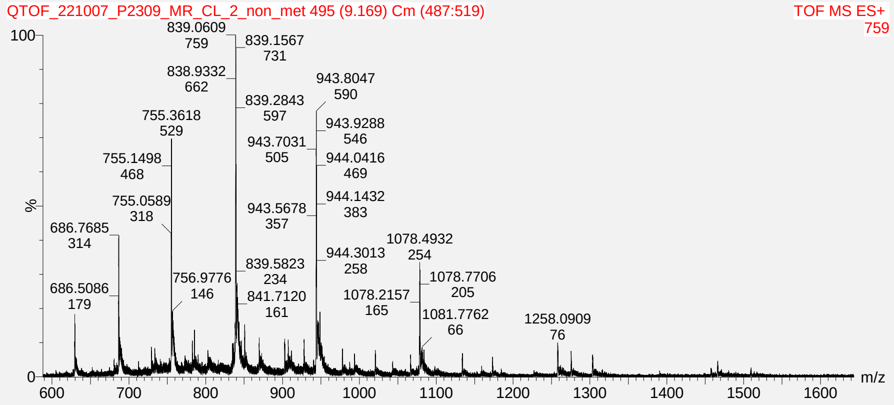

Measured Mass:

- 7543 Da
- 10197 Da
- 11723 Da

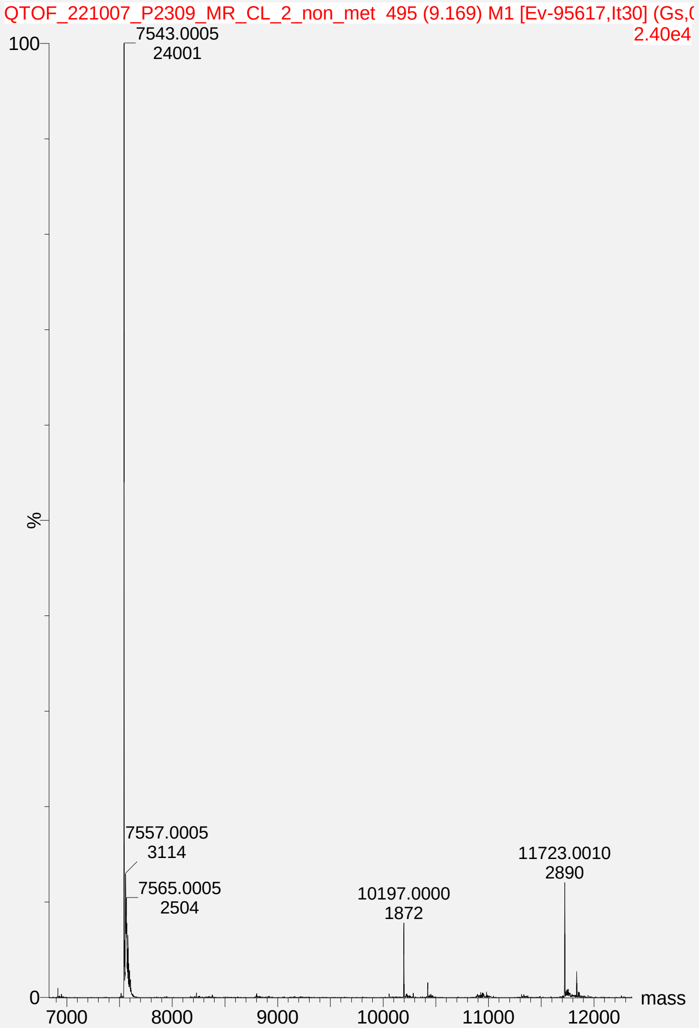

EMBL

Spectrum and Mass – Peak2

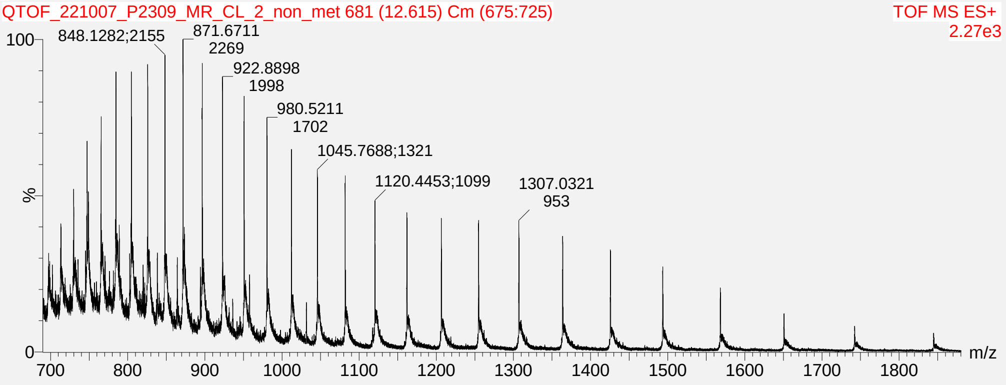

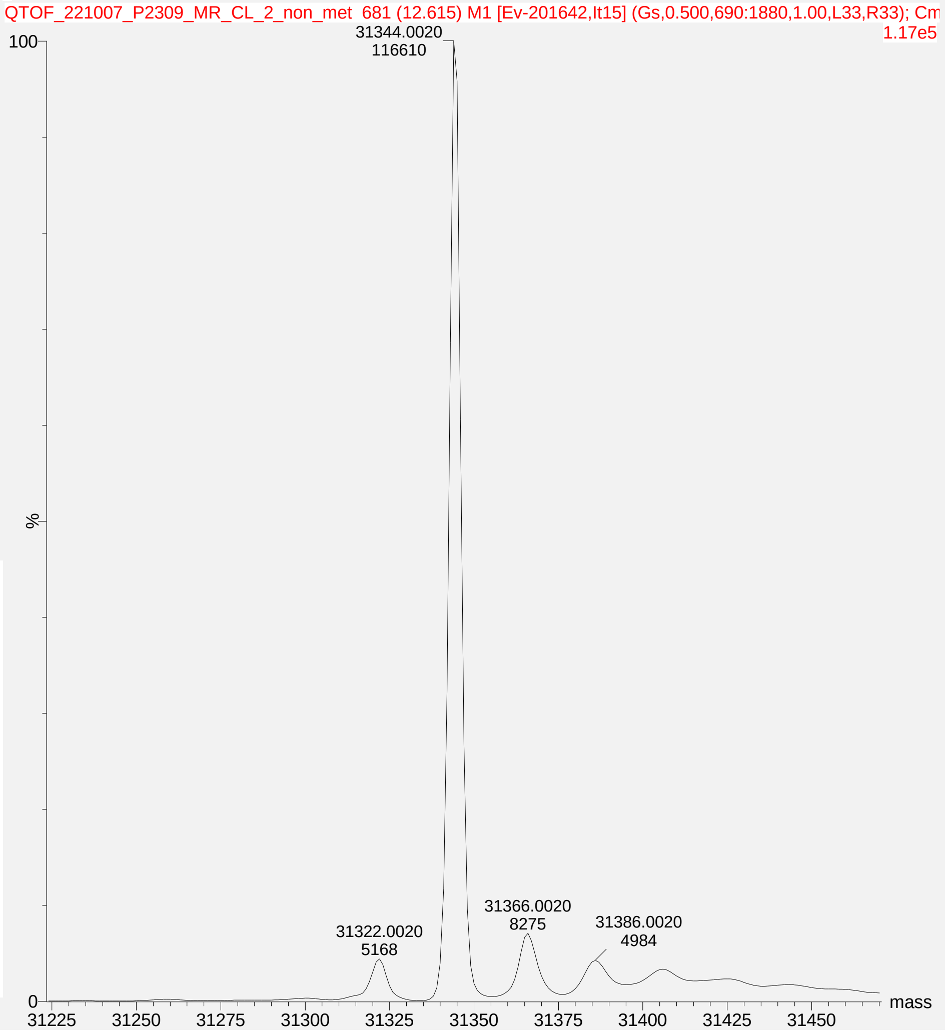

Expected Mass: 29163.54 Da
Measured Mass: 31344 Da

Compute pI/Mw

Theoretical pI/Mw (average) for the user-entered sequence:

```
        10         20         30         40         50         60
MQKAVEITYN GKTLRGMMHL PDDVKGKVPM VIMFHGFTGN KVESHFIFVK MSRALEKVGI

        70         80         90        100        110        120
GSVRFDFYGS GESDGDFSEM TFSSELEDAR QILKFVKEQP TTDPERIGLL GLSMGGAIAG

       130        140        150        160        170        180
IVAREYKDEI KALVLWAPAF NMPELIMNES VKQYGAIMEQ LGFVDIGGHK LSKDFVEDIS

       190        200        210        220        230        240
KLNIFELSKG YDKKVLIVHG TNDEAVEYKV SDRILKEVYG DNATRVTIEN ADHTFKSLEW

       250
EKKAIEESVE FFKKELLKG
```

Theoretical pI/Mw: 5.40 / 29163.54

Intact mass measurement
P2309 Sample 2 methylated

Chromatogram

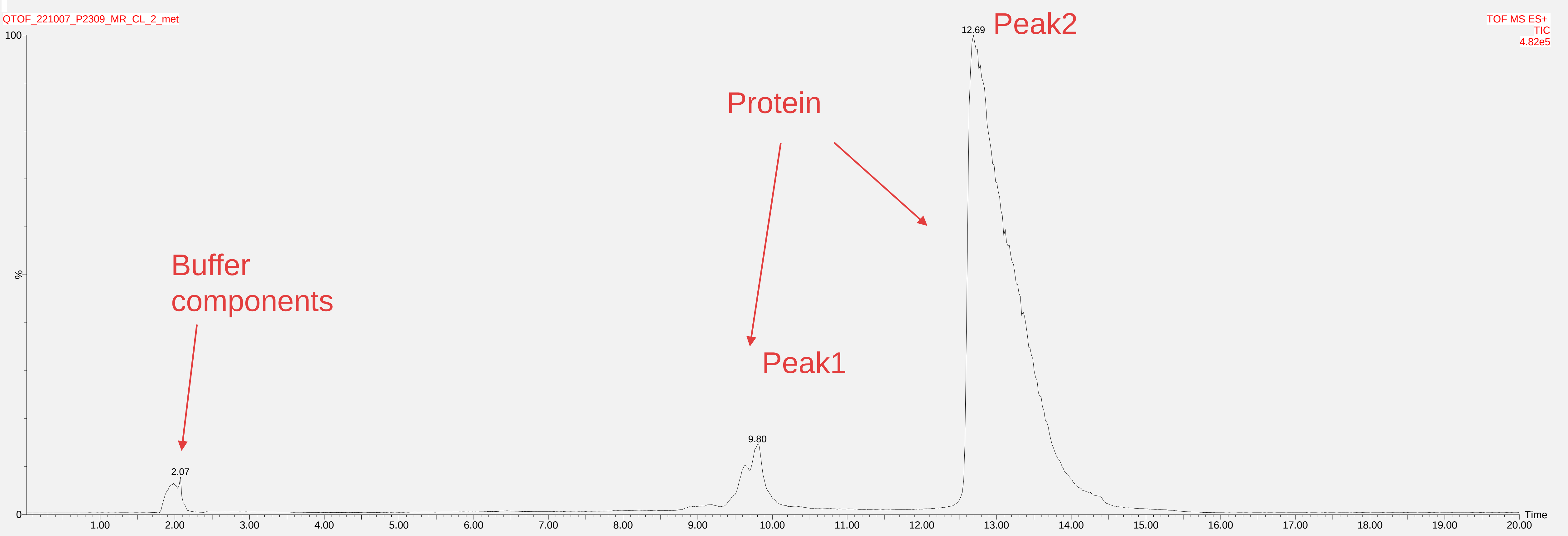

EMBL

Spectrum and Mass – Peak1

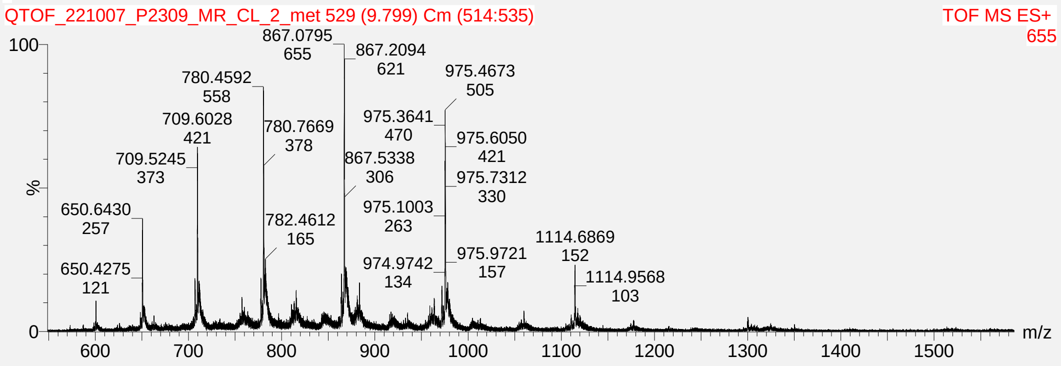

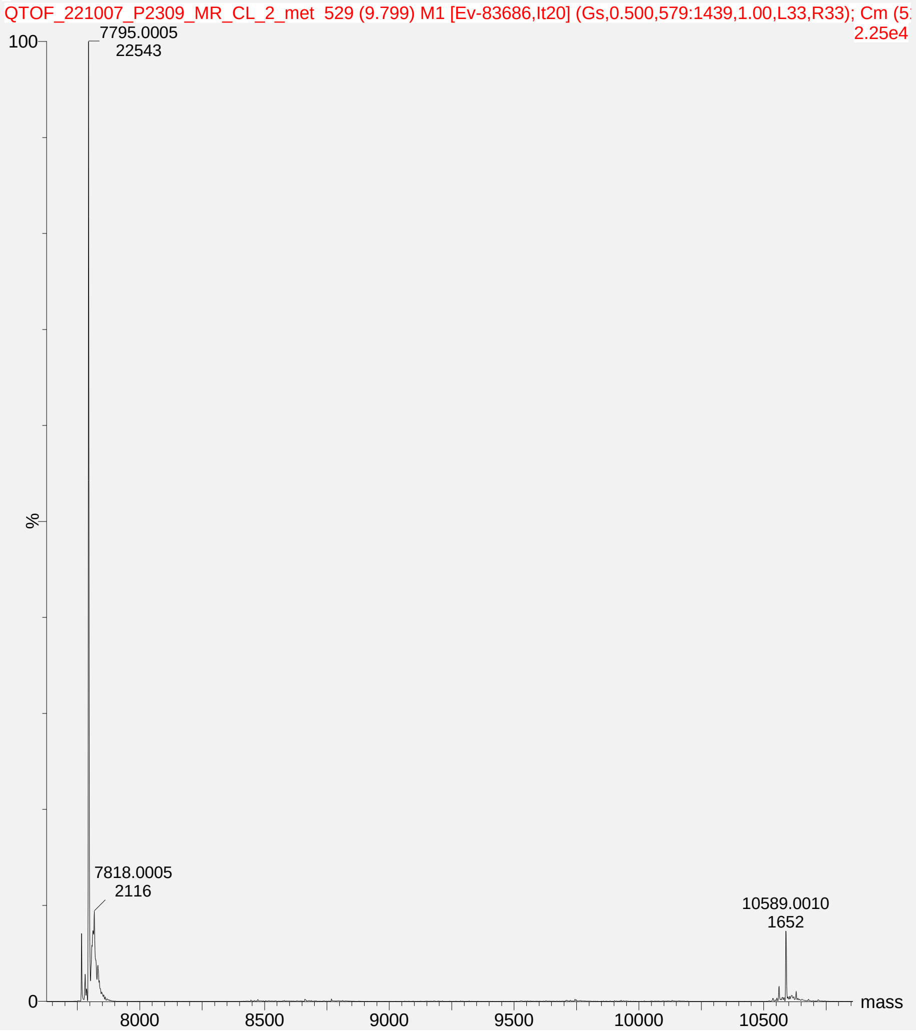

Measured Mass:

- 7542 Da
- 10197 Da

Spectrum and Mass – Peak2

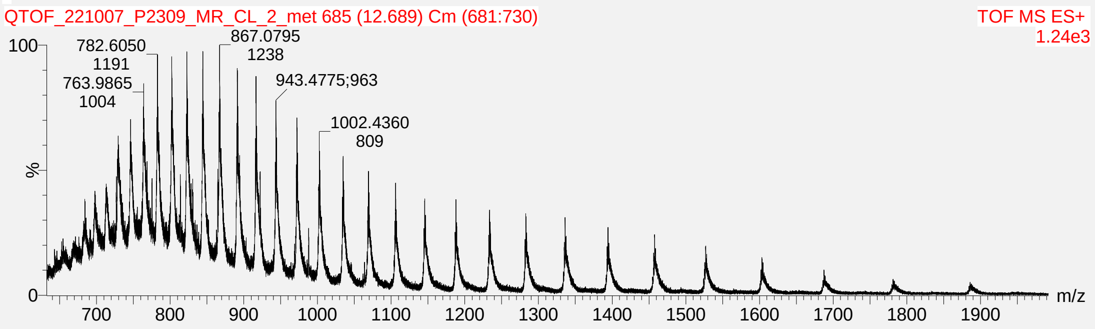

Expected Mass: 29163.54 Da
Measured Mass of non-modified: 31344 Da
Measured Mass:

- 32018 Da (+674 Da)
- 32046 Da (+702 Da)
- 32073 Da (+729 Da)

Difference: 28 Da

Difference: 27 Da

maybe a di-methylation pattern

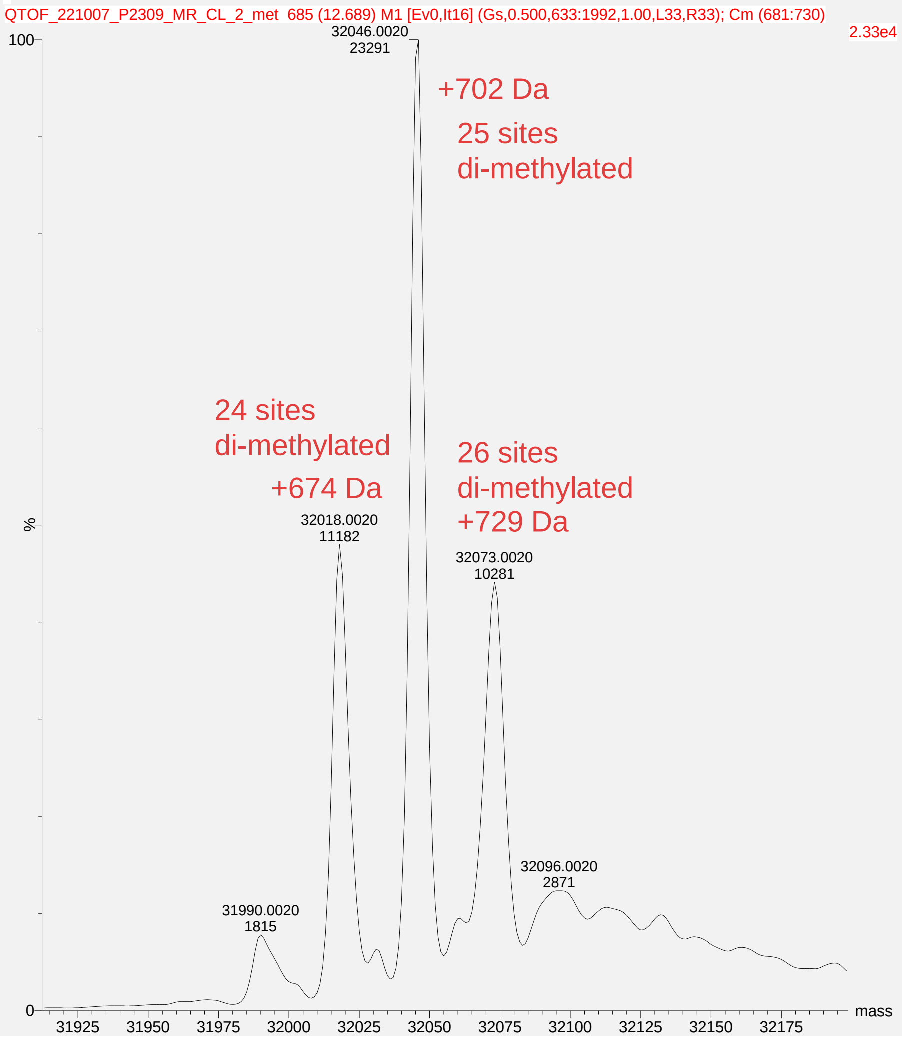

EMBL
